# Early socially isolated rats of both sexes reduce alcohol consumption and seeking by the Galanin (1–15) and naltrexone combination

**DOI:** 10.3389/fphar.2026.1836976

**Published:** 2026-09-18

**Authors:** Marta Flores-Gómez, Noelia Cantero-García, Juan Pedro Pineda-Gómez, Antonio Flores-Burgess, Zaida Díaz-Cabiale, Carmelo Millón

**Affiliations:** Instituto de Investigación Biomédica de Málaga, Facultad de Medicina, Universidad de Málaga, Campus de Teatinos s/n, Málaga, Spain

**Keywords:** alcohol, alcohol consumption, alcohol seeking, galanin (1–15), naltrexone, social isolation

## Abstract

**Background:**

Alcohol Use Disorder (AUD) is a psychiatric condition whose severity can be amplified by social factors, such as social isolation, particularly during adolescence, which exacerbates development of AUD in adulthood. Our previous studies have demonstrated that the Galanin (1–15) fragment [GAL (1–15)] reduces alcohol seeking and consumption. Furthermore, the combination of GAL (1–15) with Naltrexone (NTX)-an approved but moderately effective treatment for AUD- potentiates reduced alcohol intake and seeking behavior, showing interaction with the opioid system.

**Objective:**

Previous studies on the effects of GAL (1–15) on AUD have been conducted mainly in male animals, leaving the effects on females largely unexplored despite the well-established sex differences in alcohol use disorder. Therefore, this study introduces early-life social isolation as a critical environmental vulnerability factor for adult alcohol abuse and evaluates these mechanisms in female subjects for the first time. Specifically, we investigate the combined effects of GAL (1–15)+NTX on alcohol consumption and seeking in male and female adolescent-isolated rats, along with associated changes in mesolimbic gene expression.

**Methods:**

In socially isolated male and female rats, we evaluated GAL (1–15)+NTX in terms of voluntary alcohol intake (two-bottle choice) and reward-seeking behavior (self-administration). We analyzed transcriptional changes in C-Fos, MOR, dopamine and galanin receptors in the ventral tegmental area and nucleus accumbens.

**Results:**

Social isolation moderately increased ethanol intake and operant alcohol responding in both sexes. GAL (1–15)+NTX significantly reduced operant responding in isolated rats of both sexes, but reduced alcohol consumption only in males, with no significant effect in females at the tested doses. These effects were associated with alterations in C-Fos, MOR and dopamine receptor expression, suggesting that there is an association between molecular changes related to the mesolimbic and opioid systems and the observed reduction in alcohol-seeking behavior and consumption, even in a model of early social isolation.

**Conclusion:**

These findings suggest that GAL (1–15)+NTX could be explored further as a potential strategy in AUD research, particularly in socially vulnerable contexts.

## 1 Introduction

Alcohol Use Disorder (AUD) is a chronic recurrent condition with a high global prevalence, characterized by impaired attention, aggressive behavior, and a significant loss of control over alcohol consumption (Koob, 2024; World Health Organization, 2024; Yaseen et al., 2024). The global impact of alcohol consumption on the health of the population account for 2.6 million deaths representing 4.7% of all deaths in the world, while in Europe, alcohol problems are responsible for more than 7% of health problems and premature deaths (World Health Organization, 2024).

Several factors contribute to alcohol consumption and abuse, including stress from social isolation, particularly early in life, which increases vulnerability to AUDs in adulthood (Wardell et al., 2020; Le et al., 2021). Alcohol is often used to cope with stress and the negative emotions stemming from loneliness (Skewes and Gonzalez, 2013), highlighting the importance of social support for individuals dealing with alcohol-related issues (Akerlind and Hörnquist, 1992). A recent situation is the COVID-19 pandemic heightened anxiety, stress, and depression, and increased alcohol consumption (Clay and Parker, 2020; Rehm et al., 2020; Charles et al., 2021; Kilian et al., 2022). Several animal studies demonstrate that social isolation, particularly during the critical developmental period of postnatal days 21–42, significantly increases alcohol intake in adulthood and enhances alcohol-induced place preference (McCool and Chappell, 2009; Whitaker et al., 2013; Butler et al., 2014; Lesscher et al., 2015). Similarly, rats raised in isolation also display greater alcohol consumption and higher operant response rates compared to socially housed rats (McCool and Chappell, 2009; Chappell et al., 2013; Lesscher et al., 2015). In addition, early social isolation induces long-lasting neurobiological alterations within the mesocorticolimbic reward circuitry including changes in dopaminergic neutrotransmission synaptic, plasticity, and stress-related signaling, which have been associated with increased vulnerability to alcohol use (McWain et al., 2022).

A significant treatment gap accompanies the high prevalence of AUDs, as many individuals fail to seek treatment, and there are currently no fully effective medications available to reduce alcohol consumption and abuse (Carvalho et al., 2019; Witkiewitz et al., 2019). Naltrexone (NTX), a mu-opioid receptor antagonist that acts by attenuating alcohol-induced opioid activity, is one of the Food Drug Administration (FDA) and European Medicines Agency (EMA)-approved drugs and is among the most recommended treatments for alcoholism. Despite reducing cravings and alcohol consumption, its effectiveness is contradictory due to issues like drug dependence and side effects (Witkiewitz et al., 2019), which underscores the need for new treatments offering a broader range of options for AUD.

Recent studies highlight the role of neuropeptides in various aspects of alcohol abuse. Galanin (GAL), a neuropeptide widely distributed in the central nervous system, has been shown to increase alcohol consumption by activating the brain’s reward circuitry, notably by enhancing dopamine release in the nucleus accumbens (NAc), a key structure within the mesocorticolimbic reward system, plays a central role in reward processing and addiction (Rada et al., 1998; Lewis et al., 2004; Rada et al., 2004; Picciotto, 2010). Three GAL receptor (GALR1-3) subtypes with high affinities for GAL have been cloned (Branchek et al., 2000; Mitsukawa et al., 2008). GALR1 and GALR3 mainly activate inhibitory G proteins Gi/Go, while GALR2 mainly couples to Gq/G11 to mediate excitatory signalling (Branchek et al., 2000). Interestingly, while GAL reduces morphine preference, it is also associated with increased vulnerability to opioid use disorder (Picciotto, 2008; Holmes et al., 2012). This may be related to the presence of MOR-GALR1 heteromers in the ventral tegmental area (VTA), a key component of the mesocorticolimbic reward circuitry, which regulate dopaminergic activation, highlighting GPCR heteromers as potential targets for drug development (Moreno et al., 2017; Cai et al., 2019; De Oliveira et al., 2022).

Not only the complete Galanin molecule but also the 1–15 fragment [GAL (1–15)] have been shown to have activity in the CNS, showing an essential role in mood disorders and alcohol use disorder (Millón et al., 2014; Millón et al., 2019; Cantero-García et al., 2022b). Unlike the complete GAL, the GAL (1–15) plays a differential role in mood disorders and alcoholism and acts preferentially through the GALR1-GALR2 heteroreceptor complexes (Borroto-Escuela et al., 2014; Millón et al., 2014; Flores-Gómez et al., 2025; Pineda-Gómez et al., 2025). The presence of specific binding sites for GAL (1–15) in the dorsal hippocampus, neocortex and striatum (Hedlund et al., 1992), which is part of mesolimbic dopamine system (Koob, 1992), suggests a role of GAL (1–15) in the circuits related to the rewarding and motivational effects of drugs of abuse. In previous studies, GAL (1–15) significantly reduced alcohol preference and consumption in rats through central mechanisms, showing a differential role with the complete GAL molecule (Millón et al., 2019). Not only in a voluntary model but also in an operant self-administration model, GAL (1–15) significantly reduces alcohol-seeking behavior as well as context-dependent alcohol relapse, showing the involvement of the mesocorticolimbic pathway in these effects (Cantero-García et al., 2022b).

It has been suggested that the combination of drugs may be a novel target for the search for new treatments (Witte et al., 2012). Indeed, the combination of GAL (1–15) with the antidepressant escitalopram (ESC) is effective in reducing alcohol seeking-behavior in an operant model (Cantero-García et al., 2022a). Moreover, in our previous work we demonstrated for the first time that the combination of NTX, a drug prescribed for AUD, together with GAL (1–15) induced a substantial reduction in alcohol-seeking behavior both in an operant self-administration model and in a voluntary two-bottle model (Flores-Gómez et al., 2025). In addition, we observed changes in the immediate-early gene C-Fos, the mu-opioid receptor (MOR), and dopamine receptors in the ventral tegmental area (VTA) and the nucleus accumbens (NAc), so the mesolimbic circuit was involved in behavior mediated by GAL (1–15)+NTX (Flores-Gómez et al., 2025).

Considering these factors, the present study introduces early-life social isolation as a critical environmental vulnerability factor for adult alcohol abuse and evaluates its underlying mechanisms in female subjects for the first time. Based on previous evidence, we hypothesized that the combination of GAL (1–15) and NTX would attenuate alcohol-related behaviors in socially isolated rats and would be associated with changes in the mesocorticolimbic reward pathway. Specifically, we aimed to investigate the therapeutic potential of the GAL (1–15) and NTX combination on voluntary alcohol consumption using a two-bottle choice model, as well as on motivation and alcohol-seeking behavior using an operant self-administration paradigm in both male and female adolescent-isolated rats. Furthermore, to explore the neurobiological mechanisms involved, we analyzed whether the GAL (1–15)+NTX combination modulates the mesolimbic pathway by evaluating mRNA expression of c-Fos, mu-opioid receptors (MOR), dopamine receptors, galanin receptors, and 5-HT1A receptors in the ventral tegmental area and nucleus accumbens via RT-qPCR.

## 2 Materials and methods

### 2.1 Animals

Adolescent male and female Sprague-Dawley rats (postnatal days 21–27) were purchased from Charles River (Barcelona, Spain). The animals followed a social isolation protocol in which one group of rats was housed for 6 weeks under isolated conditions in standard individual cages (42.5 × 26.6 × 18.5 cm). To prevent visual contact and minimize social stimulation from other animals, the cages were externally covered with an opaque black material, generating black-tinted individual housing conditions. The non-isolated group was housed in standard group cages (McCool and Chappell, 2009). The animals were randomly assigned to the experimental groups. Temperature and humidity were controlled, and the light/dark cycle was reversed. All procedures followed ethical guidelines from the University of Málaga’s Guidelines for the Care and Use of Laboratory Animals (Ethic Code: 22/05/2017/066). Additional details are in the Supplementary Material.

### 2.2 Materials

Naltrexone was obtained from Merck Life Science, Madrid, Spain. Galanin (1–15) was obtained from Tocris Bioscience, Bristol, United Kingdom. Solutions were prepared freshly. Galanin (1–15) was dissolved in artificial cerebrospinal fluid (aCSF; composition: 120 mM NaCl, 20 mM NaHCO_3_, 2 mM KCl, 0.5 mM KH_2_PO_4_, 1.2 mM CaCl_2_, 1.8 mM MgCl_2_, 0.5 mM Na_2_SO_4_, and 5.8 mM D-glucose, pH 7.4). Naltrexone hydrochloride was dissolved in sterile 0.9% NaCl.

### 2.3 Experimental design

#### 2.3.1 Two experimental procedures were carried out

##### 2.3.1.1 Experiment 1

Assessment of alcohol consumption and alcohol-seeking behavior in isolated male Sprague-Dawley rats.

After the isolation period, rats underwent the light-dark test to assess the effects of social isolation on anxiety-related behavior (McCool and Chappell, 2009; Millón et al., 2014) (see Supplementary Material). All animals were housed individually for the remainder of the study and underwent the two-bottle choice test and the alcohol self-administration test (McCool and Chappell, 2009; Cantero-García et al., 2022b; Flores-Gómez et al., 2025) (see Supplementary Material). After the final alcohol self-administration test, the brains of the animals were removed for qPCR analysis.

##### 2.3.1.2 Experiment 2

Assessment of alcohol consumption and alcohol-seeking behavior in isolated female Sprague-Dawley rats.

After the isolation period, rats were tested using the Open Field Test (OFT) and Elevated Plus Maze (EPM) (Cantero-García et al., 2022a; Flores-Gómez et al., 2025) (see Supplementary Material) to evaluate the effects of social isolation on anxiety-related behavior. Subsequently, all animals were individually housed for the remainder of the study and half of the cohort was assigned to assess alcohol consumption using the two-bottle choice, while the other half was used to evaluate alcohol-seeking behavior in the alcohol self-administration (Millón et al., 2019; Cantero-García et al., 2022b; Flores-Gómez et al., 2025). After the final alcohol self-administration test, the brains of the animals were removed for qPCR analysis.

The time between injections in the animals exceeded 1 month between the two-bottle choice test and the alcohol self-administration test.

Female estrous cycles were monitored by daily vaginal cytology throughout the study, as described previously (Byers et al., 2012). Behavioral testing was performed only during the metestrus-diestrus phase to minimize hormonal variability and avoid estrus-related effects, as fluctuations in ovarian hormones during the estrous cycle have been shown to influence behavioral responses, including reward- and anxiety-related behaviors (Marcondes et al., 2001; Yoest et al., 2014) and ensure the consistency of the experimental conditions (Becker et al., 2005).

Accordingly, behavioral testing was postponed until all female rats had reached the appropriate estrous phase.

### 2.4 Behavioral assessment

#### 2.4.1 Two-bottle choice test

To analyze the effects of social isolation and pharmacological treatments on alcohol intake, male and female rats were tested using the two-bottle choice paradigm as previously described (McCool and Chappell, 2009; Millón et al., 2019). All male and female rats initially performed a two-bottle choice training (see the Supplementary Material for details). Subsequently, ethanol intake was compared between isolated and non-isolated rats to assess isolation effects. During the days preceding the experiment, the animals were handled daily to accustom them to the experimenter and the injection procedure, thereby minimising the stress associated with experimental handling.

Afterwards, isolated rats were treated with the combination of GAL (1–15) and NTX to evaluate its effect on ethanol intake. For the experiment 1, groups of male isolated rats received a subcutaneous (SC) injection of NTX 1 mg/kg or saline 30 min before the test and an intracerebroventricular injection (ICV) of GAL (1–15) 1 nmol or aCSF, 15 min before the test (Flores-Gómez et al., 2025). This resulted in four experimental groups: Saline and aCSF, saline and GAL (1–15), NTX and aCSF, NTX and GAL (1–15). For the experiment 2, two procedures were conducted in female isolated rats to assess pharmacological effects. The first evaluated dose-response effects of ICV GAL (1–15) (1, 3, 6 nmol) or aCSF 15 min before the test, and SC NTX (1, 3, 6 mg/kg) or saline 30 min before the test. The second test combined the effects of NTX 1 mg/kg or saline and GAL (1–15) 1 nmol or aCSF. Each animal received only one drug treatment, and comparisons were made between independent groups.

Ethanol and water intake and ethanol preference were measured at 2 h.

#### 2.4.2 Alcohol self-administration test

To evaluate alcohol seeking, rats were trained to self-administer 10% alcohol in 30-min sessions on an FR1 schedule until stable intake was achieved, using a modified self-administration procedure (Cantero-García et al., 2022b; Flores-Gómez et al., 2025) (see Supplementary Material). During the days preceding the experiment, the animals were handled daily to accustom them to the experimenter and the injection procedure, thereby minimising the stress associated with experimental handling.

Initially, to evaluate the impact of social isolation on alcohol-seeking behavior, isolated and non-isolated male and female rats were tested using operant procedures assessing motivation and alcohol seeking using the self-administration test as previously described (Cantero-García et al., 2022b) (see Supplementary Material).

Subsequently, the effects of the combination of GAL (1–15) and NTX on alcohol-seeking behavior in socially isolated rats were evaluated. For experiment 1, male isolated rats received SC NTX (0.3 mg/kg) or saline 30 min before the test and ICV GAL (1–15) (0.3 nmol) or aCSF 15 min before the test. This resulted in four experimental groups: Saline and aCSF, NTX and aCSF, saline and GAL (1–15), NTX and GAL (1–15). For experiment 2, two procedures in female isolated rats tested different doses of ICV GAL (1–15) (0.1 and 0.3 nmol) or aCSF 15 min before the test and SC NTX (0.3, 1, and 3 mg/kg) or saline 30 min before the test. Finally, the combined effects of NTX (0.3 mg/kg) and GAL (1–15) (0.1 nmol) were assessed before testing. Each animal received only one drug treatment, and comparisons were made between independent groups.

Active and inactive lever presses, as well as the number of alcohol reinforcements, were recorded.

### 2.5 Genes expression in *VTA* and *NAc*

Male and female isolated rats from experiments 1 and 2 of the alcohol self-administration test were euthanized 1 h after treatment. Brains were rapidly removed and frozen for later analysis.

Nuclei dissection, RNA isolation, and RT-qPCR were performed as previously described (Flores-Gómez et al., 2025) with modifications (see Supplementary Material). Primer sequences for C-Fos, MOR, D1, D2, GALR1, GALR2, GALR3, and 5HT1A are also provided in the Supplementary Material.

The experimental designs for all experiments are presented in the corresponding figures of the manuscript.

### 2.6 Statistical and data analysis

Data are presented as the mean ± standard error of the mean. All data were analyzed using GraphPad PRISM 9.0 (GraphPad Software, San Diego, CA, United States). To compare two experimental conditions, unpaired one-tailed Student’s t-tests were performed based on the expected directional effects of social isolation on alcohol consumption and alcohol-seeking behaviors described in previous studies. A one-way analysis of variance (ANOVA) was performed to compare more than two groups. When the overall ANOVA was statistically significant, Newman-Keuls multiple comparison *post hoc* tests were performed. Differences were considered statistically significant at p ≤ 0.05 (*p ≤ 0.05, **p < 0.01, ***p < 0.001).

## 3 Results

### 3.1 Socially isolated male rats showed increased ethanol intake and alcohol motivation as compared to non-isolated animals

Following 6 weeks of social isolation, male rats were tested with several alcohol-related assessments. During voluntary two-bottle choice training, isolated male rats showed a slight increase in alcohol consumption over the course of the second day (Student’s t-test, t_46_ = 1.87; p = 0.033; Supplementary Figure S1) and the third day (Student’s t-test, t_46_ = 1.68; p = 0.049; Supplementary Figure S1) of forced access to 10% ethanol compared to the non-isolated group. On the day of the two-bottle choice test, isolated animals exhibited a significant increase in ethanol intake compared to non-isolated animals (Student’s t-test, t_13_ = 3.50; p = 0.002; Figure 1A). Moreover, motivation for alcohol was assessed by operant self-administration, with a higher breaking point observed in the isolated group of animals compared to the non-insolated group (Student’s t-test, t_11_ = 1.85; p = 0.046; Figure 1B) and, therefore, showed a significantly higher presses during the progressive ratio session presses (Student’s t-test, t_11_ = 1.90; p = 0.042; Figure 1C).

**FIGURE 1:**
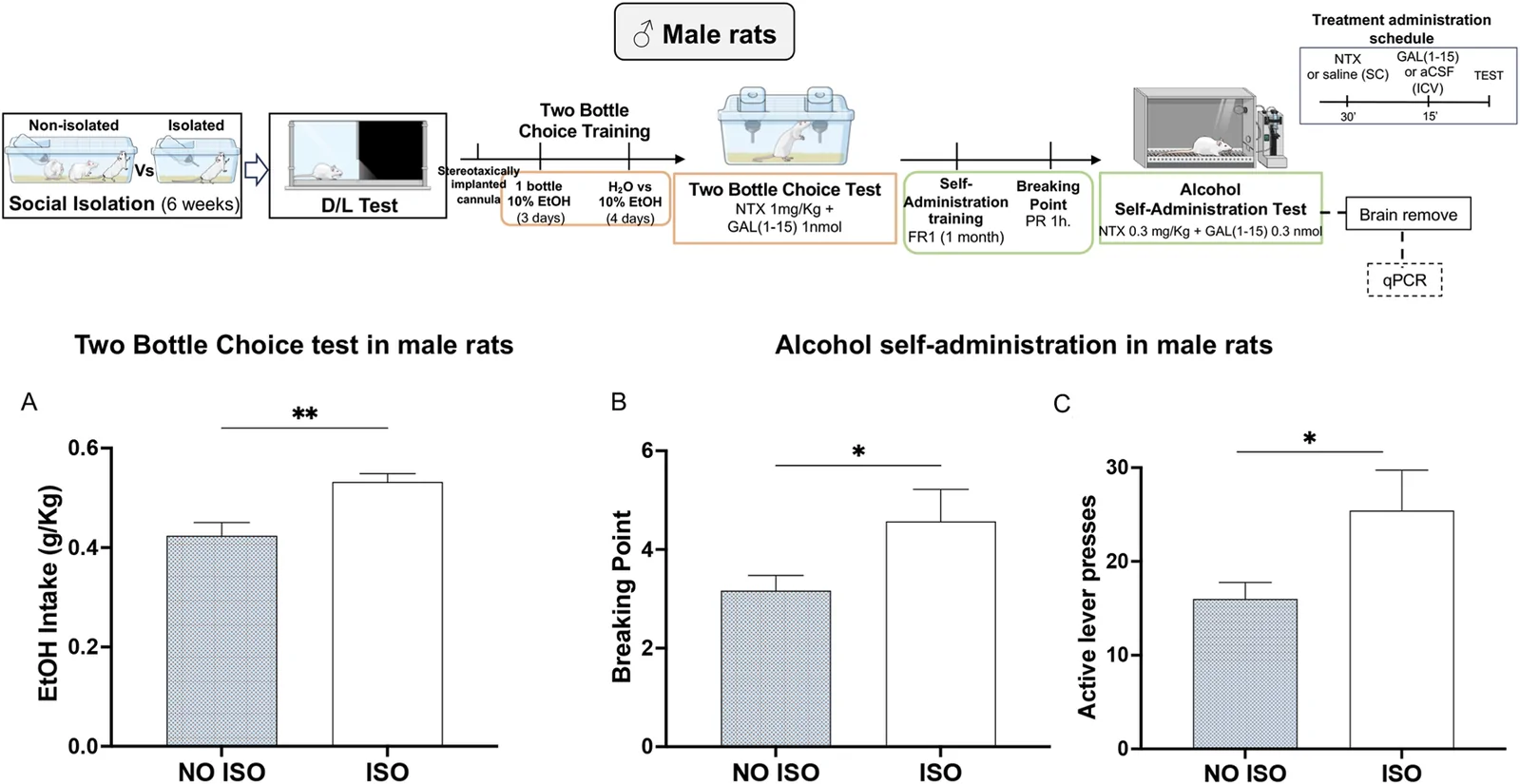
Effect of social isolation on male Sprague-Dawley rats. **(A)** Effect in the two-bottle choice paradigm between NO ISO and ISO rats. Vertical bars represent a mean ± standard error of the mean of EtOH intake (g/kg) 2 h after the test (n = 7–8 animals/group). **p < 0.01 NO ISO vs. ISO groups. **(B, C)** Effect on alcohol motivation in the self-administration test between non-isolated and isolated rats. Vertical bars represent a mean ± standard error of the mean of the breaking point and active lever presses. (n = 6–7 animals/group). *p ≤ 0.05 NO ISO vs. ISO groups during the test period. All comparisons follow a unpaired one-tailed Student’s t-test. Abbreviations: NO ISO = non-isolated; ISO = socially isolated; EtOH = ethanol.

Additionally, the state of anxiety was evaluated in the light-dark test, where no significant differences were observed between the experimental groups (Supplementary Table S2).

### 3.2 GAL (1–15)+NTX combination was associated with reduced alcohol consumption and alcohol-seeking behavior in voluntary and operant models of socially isolated male rats

#### 3.2.1 The combination of GAL (1–15)+NTX induced a significant decrease in ethanol intake on the two-bottle choice test

In the two-bottle choice test, the doses of GAL (1–15) (1 nmol) and NTX (1 mg/kg) were selected based on previous studies from our group (Flores-Gómez et al., 2025). The administration of GAL (1–15) (1 nmol) and NTX (1 mg/kg) alone groups in isolated rats did not affect ethanol intake as previously described (Figure 2A). However, the coadministration of GAL (1–15) and NTX in male isolated rats was associated with a significant decrease in ethanol intake (one-way ANOVA, F_3,25_ = 3.060, p = 0.047, p ≤ 0.05; Figure 2A) at 2 h compared to the other treatments groups.

**FIGURE 2:**
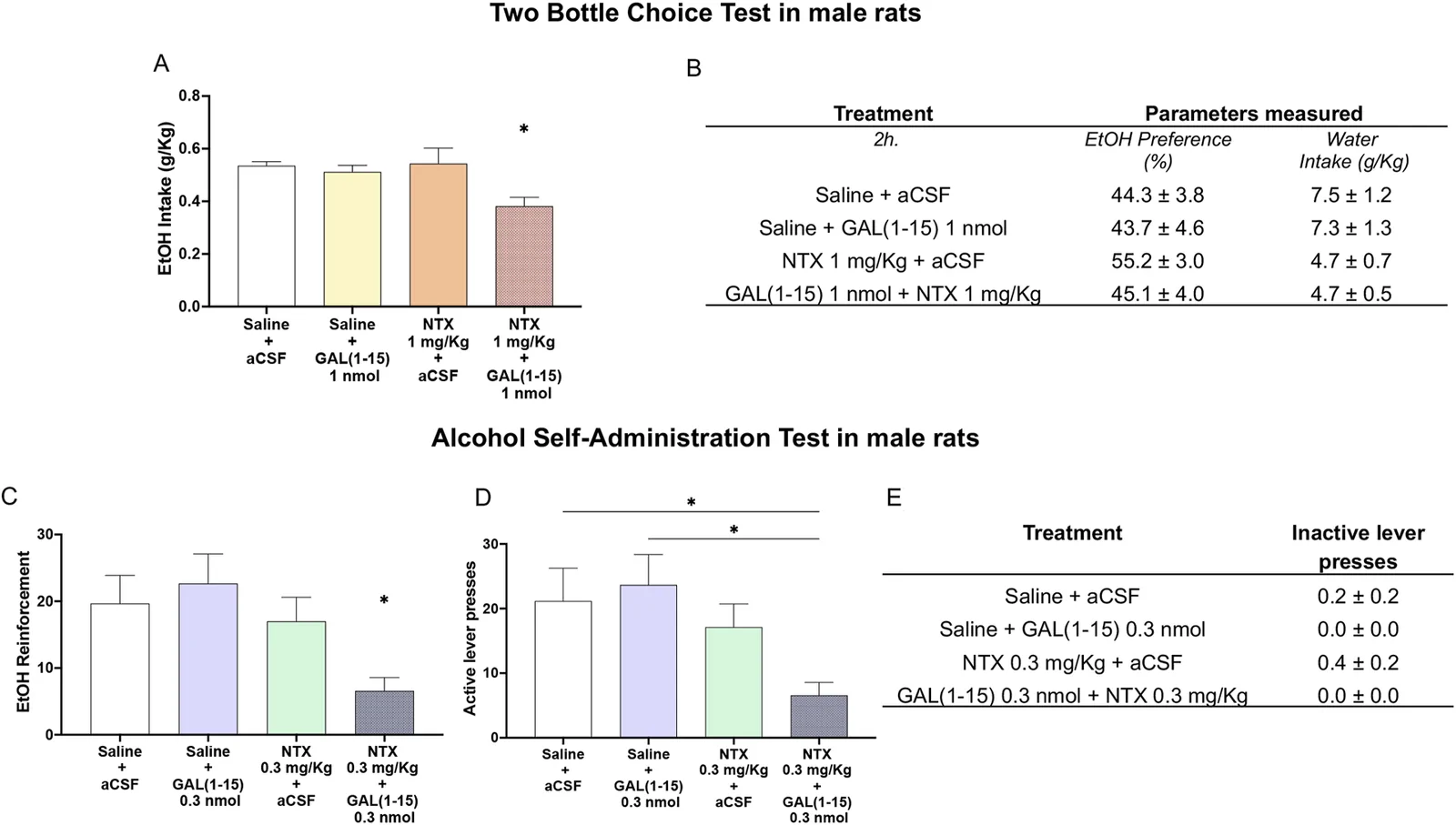
GAL (1–15) and NTX administration effect on alcohol consumption and alcohol-seeking behavior in a social isolation model in male Sprague-Dawley rats. NTX 0.3 or 1 mg/kg or saline was administrated SC 30 min before the test, and GAL (1–15) 0.3 or 1 nmol or aCSF was administered ICV 15 min before the test. (n = 5–8 animals/group). **(A)** Vertical bars represent a mean ± standard error of the mean of EtOH intake (g/kg) 2 h after the two-bottle choice test. *p ≤ 0.05 vs. the rest of the groups. **(B)** Data represent a mean ± standard error of the mean of EtOH preference (%) and water intake (g/kg) 2 h after the two-bottle choice test. There are no differences between the experimental groups. **(C, D)** Vertical bars represent a mean ± standard error of the mean of the number of alcohol reinforcements (*p ≤ 0.05 vs. the rest of the groups) and active lever presses (*p ≤ 0.05 NTX 0.3 mg/kg + GAL (1–15) 0.3 nmol group vs. saline + GAL (1–15) 0.3 nmol and saline + aCSF) in the alcohol self-administration test. **(E)** Data represent a mean ± standard error of the mean of inactive lever presses in the alcohol self-administration test. There are no differences between the experimental groups. All comparisons follow a one-way ANOVA followed by the Newman-Keuls multiple comparisons test. Abbreviations: GAL (1–15) = Galanin (1–15); NTX = naltrexone; SC = subcutaneous; aCSF = artificial cerebrospinal fluid; ICV = intracerebroventricular; EtOH = ethanol.

No differences were observed in ethanol preference or water intake 2 h after the administration of the GAL (1–15)-NTX combination in male isolated rats (Figure 2B).

#### 3.2.2 The combination of GAL (1–15)+NTX significantly reduced alcohol-seeking behavior in the alcohol self-administration test

Based on doses used in previous studies (Flores-Gómez et al., 2025), in isolated male rats, the combination of GAL (1–15) (0.3 nmol) and NTX (0.3 mg/kg) at threshold doses significantly reduced the number of alcohol reinforcers (one-way ANOVA, F_3,23_ = 3.720, p = 0.026, p ≤ 0.05; Figure 2C) compared to the remaining groups, and the number of active lever presses (one-way ANOVA, F_3,23_ = 3.720, p = 0.03, p ≤ 0.05; Figure 2D) compared with GAL (1–15)-alone, and control groups.

The GAL (1–15) and NTX groups, when injected separately, had no effects on the number of reinforcers for alcohol self-administration and the number of active lever presses (Figures 2C,D).

The total number of inactive lever presses did not significantly differ between groups (Figure 2E).

### 3.3 Socially isolated female rats showed increased ethanol intake and alcohol seeking-behavior as compared to non-isolated animals

Groups of isolated female rats were challenged with voluntary and operant models of alcohol consumption and seeking. Firstly, isolated and non-isolated female rats were trained for alcohol consumption over several days (Supplementary Figure S2). In the two-bottle choice test, the isolated group had a higher ethanol intake compared with the non-isolated group (Student’s t-test, t_18_ = 1.89; p = 0.038; Figure 3A). Moreover, in the alcohol self-administration test, the isolated group showing an increased number of alcohol reinforcements obtained by the isolated female rats compared to the non-isolated group (Student’s t-test, t_12_ = 2.31; p = 0.020; Figure 3B). During the alcohol self-administration training phase prior to the test, isolated rats already showed a slight increase in EtOH reinforcements compared with non-isolated rats (Supplementary Figure S3).

**FIGURE 3:**
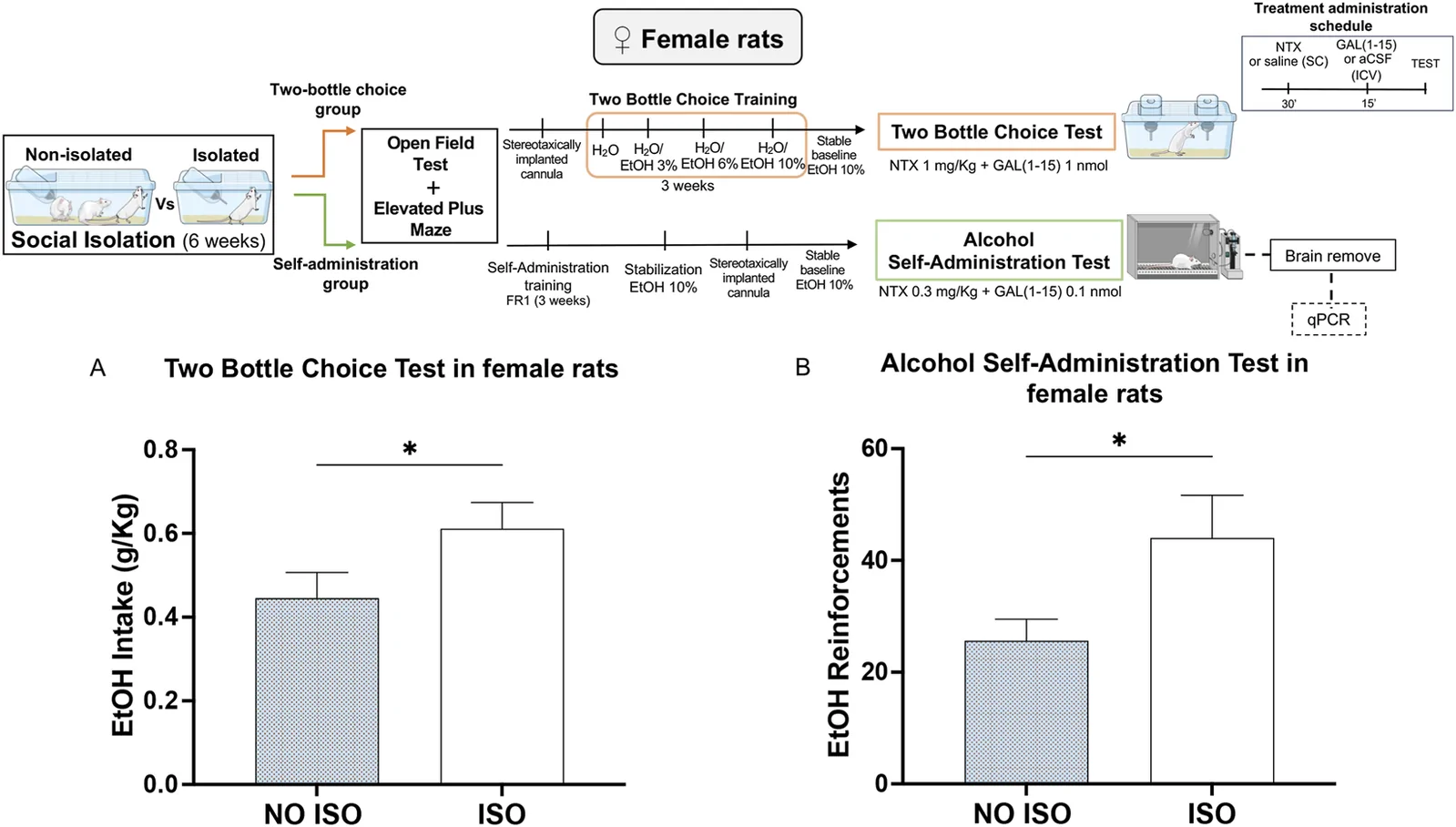
Effect of social isolation on female Sprague-Dawley rats. **(A)** Effect in the two-bottle choice paradigm between NO ISO and ISO rats. Vertical bars represent a mean ± standard error of the mean of EtOH intake (g/kg) 2 h after the test. (n = 10 animals/group). *p ≤ 0.05 NO ISO vs. ISO groups. **(B)** Effect on alcohol seeking-behavior in alcohol self-administration test between NO ISO and ISO rats. Vertical bars represent a mean ± standard error of the mean of the number of alcohol reinforcements during the test period. (n = 6–8 animals/group). *p ≤ 0.05 NO ISO vs. ISO groups. All comparisons follow a unpaired one-tailed Student’s t-test. Abbreviations: NO ISO = non-isolated; ISO = socially isolated; EtOH = ethanol.

Additionally, the state of anxiety from the isolated and the non-isolated females was evaluated with the OFT and EPM. In the OFT, the isolated group reduced the number of entries to the centre (Supplementary Table S3). In the EPM, no relevant results were obtained in the anxiety-assessed parameters (Supplementary Table S3).

### 3.4 Dose-response curve of GAL (1–15) and NTX on the two-bottle choice paradigm

Since there are no previous studies in female rats assessing alcohol consumption using GAL (1–15) and NTX, a dose-response curve was generated for both treatments. Firstly, a dose-response curve of GAL (1–15) at 1, 3 and 6 nmol in the two-bottle choice test was compiled. Among the doses tested, only GAL (1–15) at 3 nmol significantly reduced ethanol consumption by 50% at 2 h (Student’s t-test, t_10_ = 3.48; p = 0.003; Figure 4A) compared with the control group, whereas the 1 nmol and 6 nmol doses had no effect.

**FIGURE 4:**
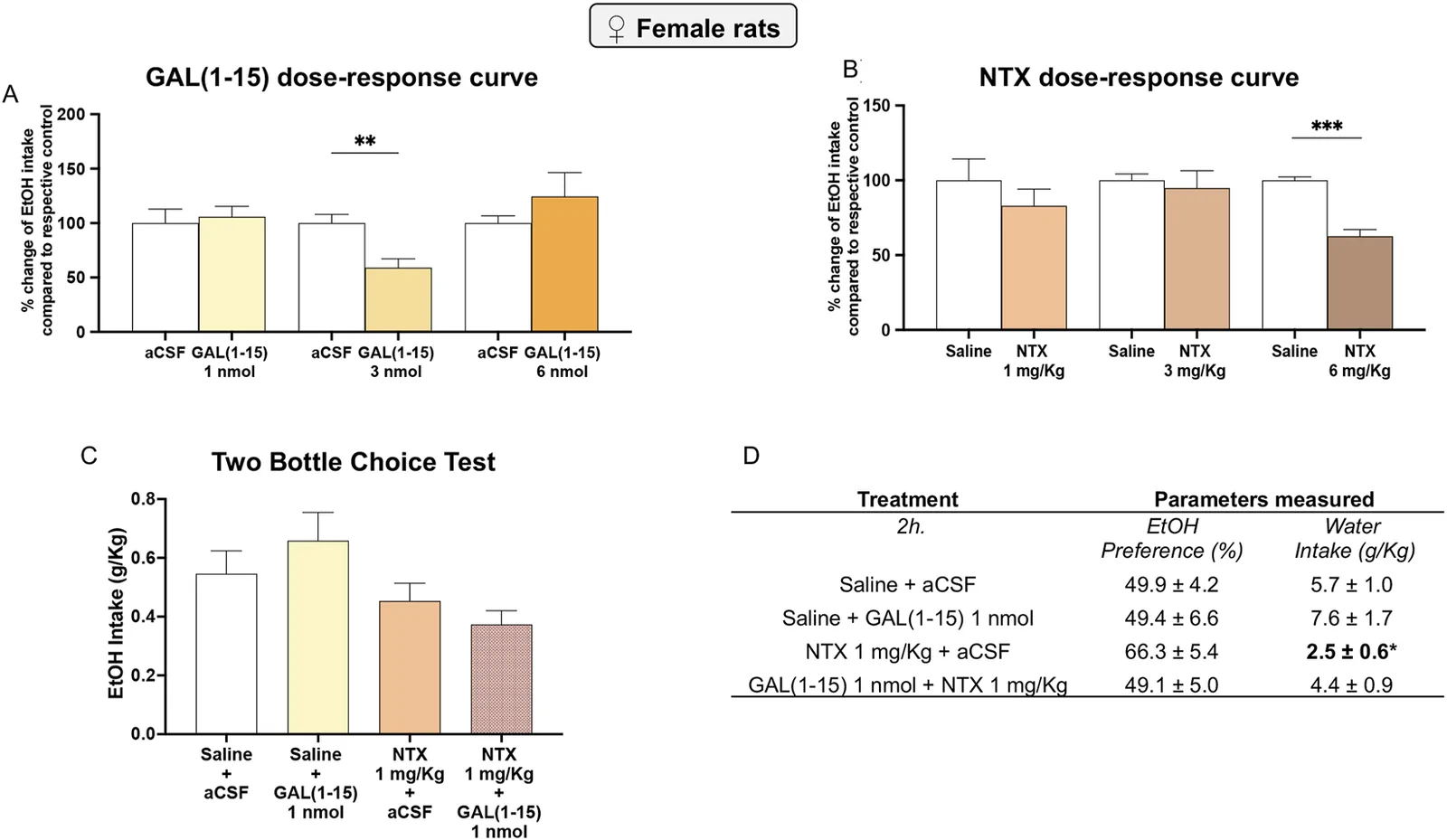
GAL (1–15) and NTX administration effect in the two-bottle choice paradigm in a social isolation model in female Sprague-Dawley rats. NTX or saline was administrated SC 30 min before the test, and GAL (1–15) or aCSF was administered ICV 15 min before the test. aCSF group (n = 6–8), GAL (1–15) 1 nmol group (n = 9), GAL (1–15) 3 nmol group (n = 5), GAL (1–15) 6 nmol group (n = 5), saline group (n = 3–7), NTX 1 mg/kg group (n = 8), NTX 3 mg/kg group (n = 6) and NTX 6 mg/kg group (n = 8). **(A)** Effect of the administration of GAL (1–15) 1, 3 and 6 nmol in the two-bottle choice test. Vertical bars represent a mean ± standard error of the mean of percent of change of EtOH intake 2 h after the test compared to the respective control. **p < 0.01 GAL (1–15) 3 nmol group vs. aCSF, according to a unpaired one-tailed Student’s t-test. **(B)** Effect of the administration of NTX 1, 3 and 6 mg/kg in the two-bottle choice test. Vertical bars represent a mean ± standard error of the mean of percent of change of EtOH intake 2 h after the test compared to the respective control. ***p < 0.001 NTX 6 mg/kg group vs. saline, according to a unpaired one-tailed Student’s t-test. Figures **(A,B)** correspond to independent experimental series conducted at different times, each with its own vehicle injection control group. Therefore, data were normalized to the corresponding control group within each experiment to allow comparison between drug concentrations. **(C)** Effect of GAL (1–15) 1 nmol and NTX 1 mg/kg combination in the two-bottle choice test. Vertical bars represent a mean ± standard error of the mean of EtOH intake (g/kg) 2 h after the test. The treatments were evaluated in the same experimental session and compared with a single shared control group. There are no differences between the experimental groups, according to a one-way ANOVA. **(D)** Data represent a mean ± standard error of the mean of EtOH preference (%) and water intake (g/kg) in the two-bottle choice paradigm 2 h after the test. Water intake: *p ≤ 0.05 vs. GAL (1–15) 1 nmol group. These comparisons follow a one-way ANOVA followed by the Newman-Keuls multiple comparisons test. Abbreviations: GAL (1–15) = Galanin (1–15); NTX = naltrexone; SC = subcutaneous; aCSF = artificial cerebrospinal fluid; ICV = intracerebroventricular; EtOH = ethanol.

In the same way, in the two-bottle choice test, isolated female rats were administered different doses of NTX (1, 3 and 6 mg/kg). The results showed that the administration of the NTX 6 mg/kg produced a significant reduction in ethanol intake compared with their control group (Student’s t-test, t_12_ = 6.73; p = 0.0001; Figure 4B), whereas the lower doses had no significant effect.

#### 3.4.1 The combination of GAL (1–15)+NTX does not modify the ethanol intake on the two-bottle choice

In the two bottle-choice test, we observed that single doses of GAL (1–15) (1 nmol) and NTX (1 mg/kg) had no effect (Figure 4C). The combination of GAL (1–15)+NTX also did not affect ethanol intake or preference at the doses used in the two-bottle choice test at 2 h (Figures 4C,D). Regarding water intake, NTX alone reduced this measure compared with the GAL (1–15)-alone group (one-way ANOVA, F_3,26_ = 3.874, p = 0.021, p ≤ 0.05; Figure 4D).

### 3.5 GAL (1–15)+NTX combination was associated with reduced alcohol-seeking behavior in the operant self-administration model of isolated female rats

Since GAL (1–15) and NTX in female rats have not been studied in self-administration, in the current work, isolated female rats were assessed to determine the threshold doses of GAL (1–15) and NTX in the alcohol self-administration test.

The results showed that GAL (1–15) at a 0.3 nmol dose induced a significant reduction in the number of reinforcements (Student’s t-test, t_13_ = 2.63; p = 0.010; Figure 5A) compared with the control group. Similarly, active lever presses were significantly reduced at the same dose (Student’s t-test, t_13_ = 2.89; p = 0.006; Supplementary Table S4). However, the dose of GAL (1–15) 0.1 nmol lacked an effect in this test (Figure 5A). No differences were observed in inactive lever presses (Supplementary Table S4).

**FIGURE 5:**
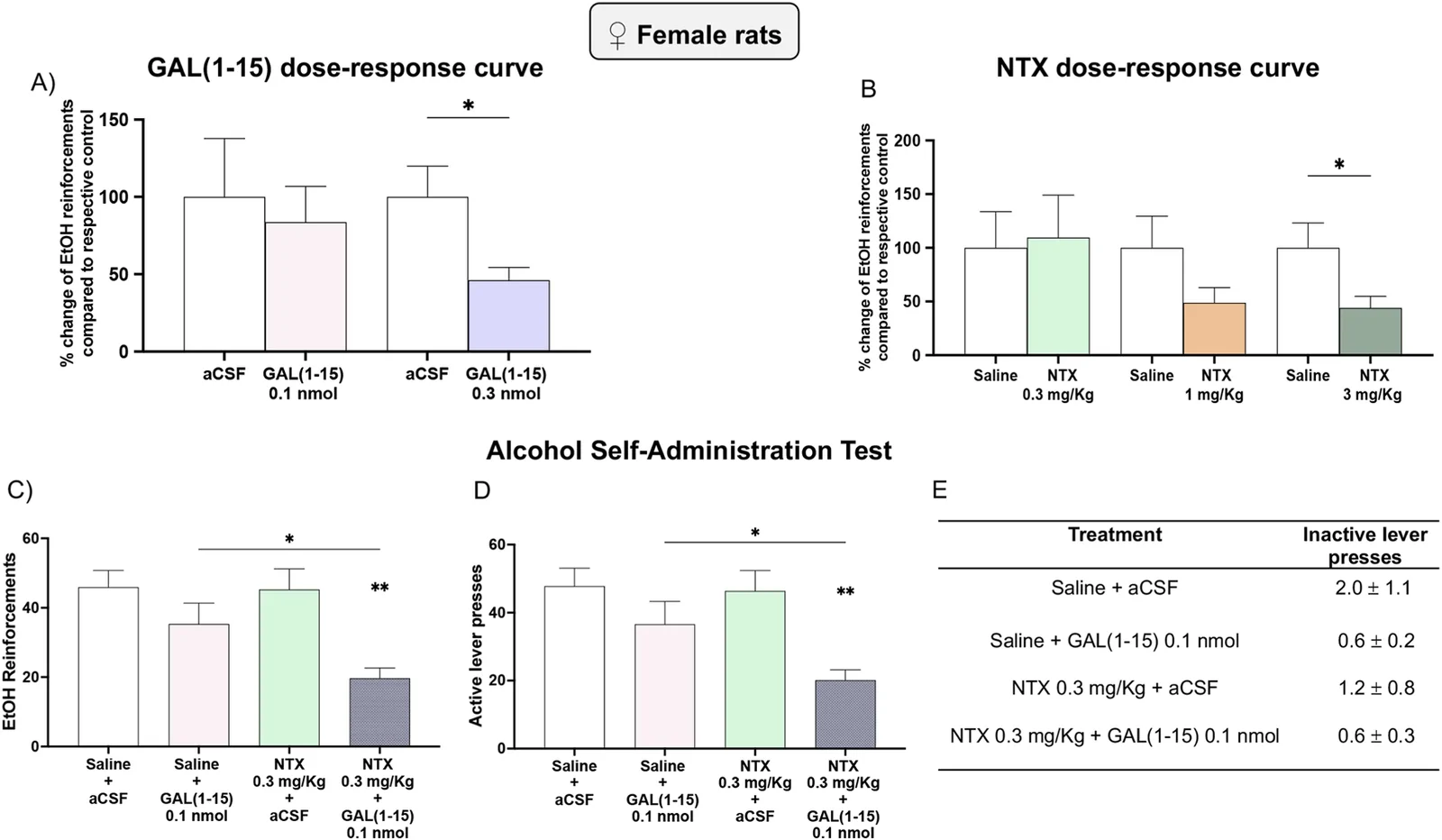
Effect of GAL (1–15) and NTX administration in the alcohol self-administration test in a social isolation model in female Sprague-Dawley rats. NTX or saline was administrated SC 30 min before the test, and GAL (1–15) or aCSF was administered ICV 15 min before the test. 4–9 animals/group. **(A)** Effect of the administration of GAL (1–15) 0.1 and 0.3 nmol in the alcohol self-administration test. Vertical bars represent a mean ± standard error of the mean of percent of change of EtOH reinforcements compared to the respective control. *p ≤ 0.05 GAL (1–15) 0.3 nmol group vs. aCSF, according to a unpaired one-tailed Student’s t-test. **(B)** Effect of the administration of NTX 0.3, 1 and 3 mg/kg in the alcohol self-administration test. Vertical bars represent a mean ± standard error of the mean of percent of change of the number of EtOH reinforcements compared to the respective control. *p ≤ 0.05 NTX 3 mg/kg group vs. saline, according to a unpaired one-tailed Student’s t-test. Figures **(A,B)** correspond to independent experimental series conducted at different times, each with its own vehicle injection control group. Therefore, data were normalized to the corresponding control group within each experiment to allow comparison between drug concentrations. **(C–E)** Effect of GAL (1–15) 0.1 nmol and NTX 0.3 mg/kg combination in the alcohol self-administration test: **(C, D)** Vertical bars represent a mean ± standard error of the mean of EtOH reinforcement and active lever presses: *p ≤ 0.05 NTX 0.3 mg/kg + GAL (1–15) 0.1 nmol group vs. GAL (1–15) 0.1 nmol group, **p < 0.01 vs. NTX 0.3 mg/kg + aCSF and saline + aCSF. **(E)** Data represent a mean ± standard error of the mean of inactive lever presses in the alcohol self-administration test. There are no differences between the experimental groups. These comparisons follow a one-way ANOVA followed by the Newman-Keuls multiple comparisons test. Abbreviations: GAL (1–15) = Galanin (1–15); NTX = naltrexone; SC = subcutaneous; aCSF = artificial cerebrospinal fluid; ICV = intracerebroventricular; EtOH = ethanol.

The effect of NTX on alcohol self-administration was evaluated at three doses (0.3, 1 and 3 mg/kg) in isolated female rats, showing that NTX at the dose of 3 mg/kg significantly decreased the number of reinforcements (Student’s t-test, t_11_ = 2.48; p = 0.015; Figure 5B), compared with their control group, whereas NTX at 0.3 and 1 mg/kg lacked an effect in the number of reinforcements in this test (Figure 5B). Similarly, active lever presses were significantly reduced at the 3 mg/kg dose, (Student’s t-test, t_11_ = 2.00; p = 0.035; Supplementary Table S5), whereas no differences were observed at the lower doses. No significant differences were observed in the number of inactive lever presses across any of the doses tested (Supplementary Table S5).

In the alcohol self-administration test, GAL (1–15) 0.1 nmol and NTX 0.3 mg/kg alone groups did not affect the number of reinforcements or active lever presses (Figures 5C,D). However, the coadministration of GAL (1–15)+NTX at threshold doses in isolated females induced a significantly reduced the number of reinforcements (one-way ANOVA, F_3,32_ = 5.78, p = 0.003; Figure 5C), and active lever presses (one-way ANOVA, F_3,32_ = 5.51, p = 0.004; Figure 5D) compared with the control, NTX-alone (p < 0.01) and GAL (1–15)-alone groups (p ≤ 0.05).

GAL (1–15)+NTX combination had no effect on the number of inactive lever presses in the alcohol self-administration test (Figure 7E).

### 3.6 The combination of GAL (1–15)+NTX was associated with changes in the mRNA expression of C-Fos, MOR, dopaminergic genes, and GAL receptors in VTA and NAc, in socially isolated male and female rats

#### 3.6.1 GAL (1–15)+NTX combination increased the gene expression of C-Fos and MOR in the VTA in male and female isolated rats

In isolated male rats, the coadministration of GAL (1–15)+NTX at subthreshold doses significantly increased C-Fos expression in VTA (one-way ANOVA, F_3,20_ = 5.43, p = 0.007; Figure 6A) compared to the control (p ≤ 0.05), GAL (1–15) and NTX groups (p < 0.01). In the same way, in isolated female rats, only the group treated with the combination of GAL (1–15)+NTX showed a significant increase in C-Fos expression (one-way ANOVA, F_3,19_ = 6.02, p = 0.005; Figure 6B), compared with the control, GAL (1–15) (p ≤ 0.05) and NTX groups (p < 0.01).

**FIGURE 6:**
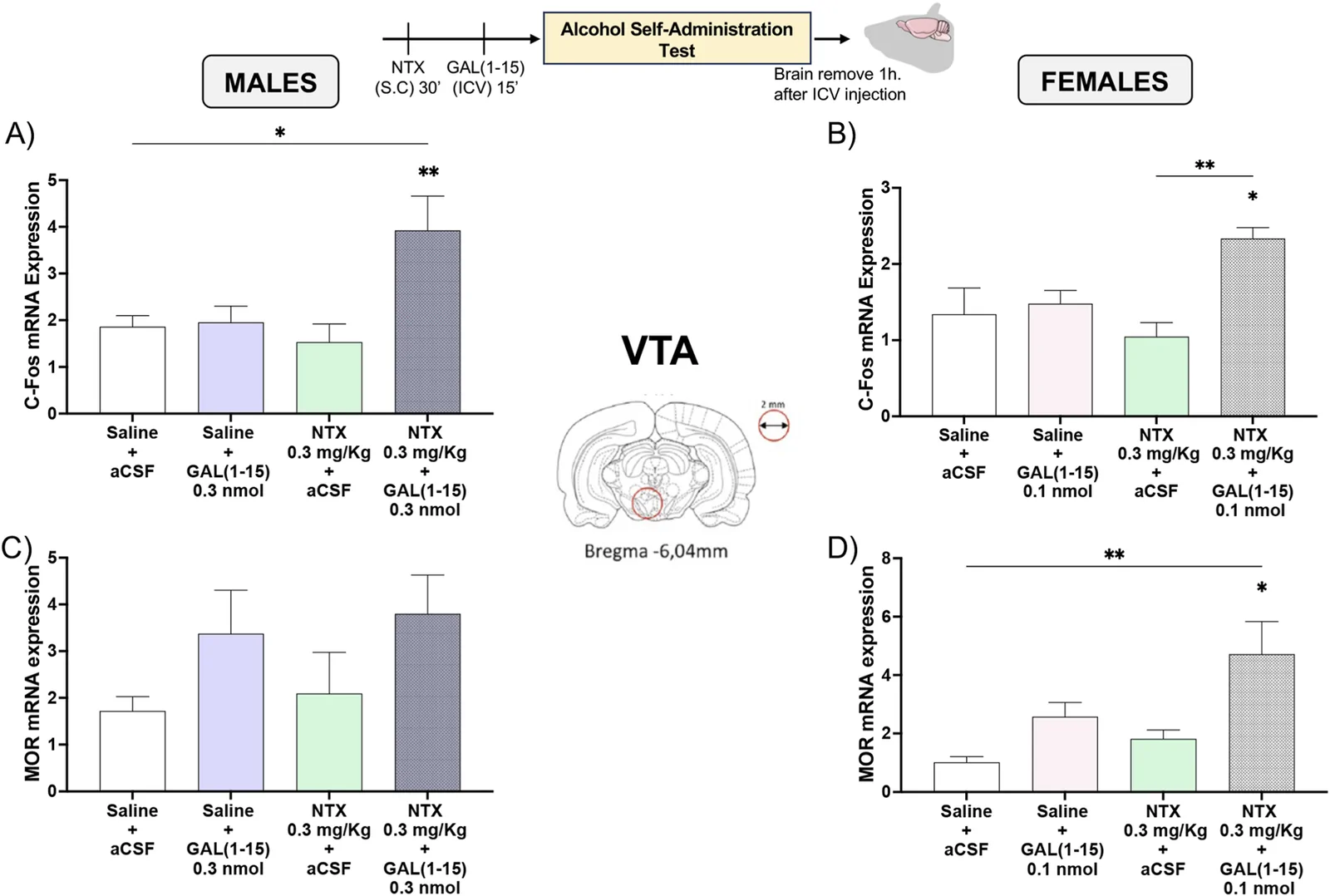
Effect of the combination of GAL (1–15) and NTX on C-Fos and MOR mRNA expression in the ventral tegmental area (VTA) in a social isolation model in male and female Sprague-Dawley rats. Measures were taken 1 h after ICV injection. Male and female rats: saline + aCSF (n = 5–6), GAL (1–15) group (n = 5–6), NTX group (n = 5–6), and GAL (1–15)+NTX group (n = 5–6). Vertical bars represent the mean ± standard error of the mean of mRNA expression of: **(A)** C-Fos in male rats. *p ≤ 0.05 NTX 0.3 mg/kg + GAL (1–15) 0.3 nmol group vs. saline + aCSF; **p < 0.01 vs. saline + GAL (1–15) 0.3 nmol and NTX 0.3 mg/kg + aCSF groups. **(B)** C-Fos in female rats. *p ≤ 0.05 vs. saline + aCSF and saline + GAL (1–15) 0.1 nmol groups; **p < 0.01 NTX 0.3 mg/kg + GAL (1–15) 0.1 nmol vs. NTX 0.3 mg/kg + aCSF group. **(C)** MOR in male rats. There are no differences between the experimental groups. **(D)** MOR in female rats. *p ≤ 0.05 vs. saline + GAL (1–15) 0.1 nmol and NTX 0.3 mg/kg + aCSF groups, **p < 0.01 NTX 0.3 mg/kg + GAL (1–15) 0.1 nmol vs. saline + aCSF. All comparisons follow a one-way ANOVA followed by the Newman-Keuls multiple comparisons test. Abbreviations: GAL (1–15) = Galanin (1–15); NTX = naltrexone; MOR = µ-opioid receptor; VTA = ventral tegmental area; ICV = intracerebroventricular; aCSF = artificial cerebrospinal fluid.

**FIGURE 7:**
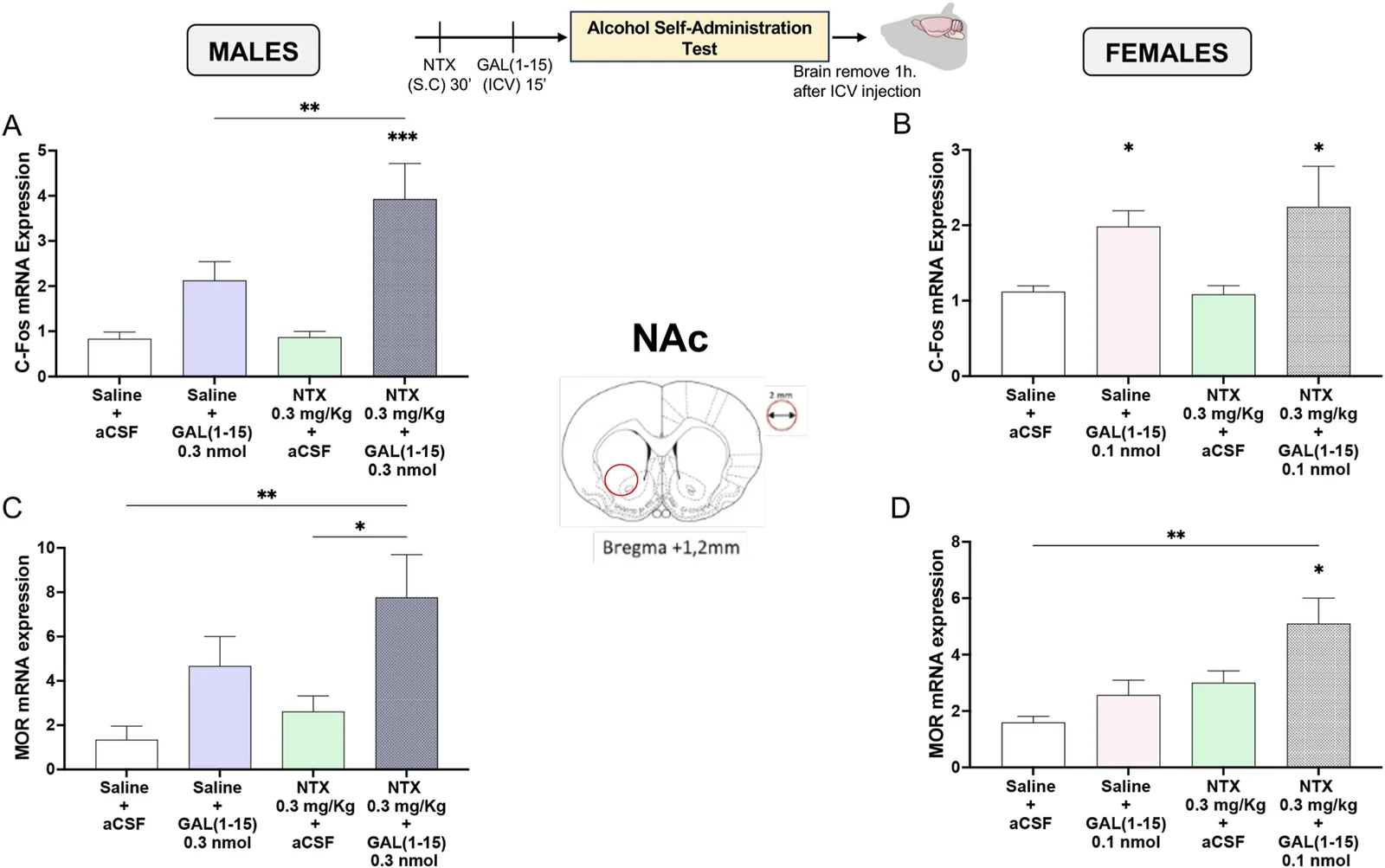
Effect of the combination of GAL (1–15) and NTX on C-Fos and MOR mRNA expression in the Nucleus Accumbens (NAc) in a social isolation model in male and female Sprague-Dawley rats. Measures were taken 1 h after ICV injection. Male and female rats: saline + aCSF (n = 4–6), GAL (1–15) group (n = 4–6), NTX group (n = 4–6), and GAL (1–15)+NTX group (n = 4–6). Vertical bars represent the mean ± standard error of the mean of mRNA expression of: **(A)** C-Fos in male rats. **p < 0.01 NTX 0.3 mg/kg + GAL (1–15) 0.3 nmol group vs. saline + GAL (1–15) 0.3 nmol group; ***p < 0.001 vs. saline + aCSF and NTX 0.3 mg/kg + aCSF groups. **(B)** C-Fos in female rats. *p ≤ 0.05 vs. saline + aCSF and NTX 0.3 mg/kg + aCSF groups. **(C)** MOR in male rats. *p ≤ 0.05 NTX 0.3 mg/kg + GAL (1–15) 0.3 nmol group vs. NTX 0.3 mg/kg + aCSF group, **p < 0.01 NTX 0.3 mg/kg + GAL (1–15) 0.3 nmol group vs. saline + aCSF. **(D)** MOR in female rats. *p ≤ 0.05 vs. saline + GAL (1–15) 0.1 nmol and NTX 0.3 mg/kg + aCSF groups, **p < 0.01 NTX 0.3 mg/kg + GAL (1–15) 0.1 nmol group vs. saline + aCSF. All comparisons follow a one-way ANOVA followed by the Newman-Keuls multiple comparisons test. Abbreviations: GAL (1–15) = Galanin (1–15); NTX = naltrexone; MOR = µ-opioid receptor; NAc = nucleus accumbens; ICV = intracerebroventricular; aCSF = artificial cerebrospinal fluid.

As shown in Figure 6C, the GAL (1–15)-alone group and the GAL (1–15)+NTX combination group showed an increase in MOR expression in the VTA of male rats, although it was not significant. However, in female rats, the combination of GAL (1–15) (0.3 nmol) and NTX (0.3 mg/kg) was associated with a significant increase in the mRNA level of MOR 1 hour after its administration (one-way ANOVA, F_3,20_ = 6.26, p = 0.004; Figure 6D) compared with the control (p < 0.01) and GAL (1–15) and NTX groups (p ≤ 0.05).

#### 3.6.2 GAL (1–15)+NTX combination increased the mRNA expression of C-Fos and MOR in the NAc in male and female isolated rats

In NAc, the coadministration of GAL (1–15) (0.3 nmol) and NTX (0.3 mg/kg) in male isolated rats significantly leads to increased C-Fos expression (one-way ANOVA, F_3,19_ = 11.48, p = 0.0002; Figure 7A) compared to control, NTX (p < 0.001) and GAL (1–15) groups (p < 0.01). In isolated female rats, GAL (1–15)+NTX also significantly increases C-Fos expression compared to control (one-way ANOVA, F_3,16_ = 5.90, p = 0.007, p ≤ 0.05; Figure 7B) and the NTX group.

In MOR mRNA expression in NAc, again, only the combination of GAL (1–15)+NTX is associated with an increase in the MOR expression, both in isolated males (one-way ANOVA, F_3,17_ = 5.43, p = 0.008; Figure 7C) compared with the control (p < 0.01) and NTX alone group (p ≤ 0.05) and in isolated female rats (one-way ANOVA, F_3,20_ = 6.69, p = 0.003; Figure 7D) compared with the control (p < 0.01), GAL (1–15) and NTX groups (p ≤ 0.05).

#### 3.6.3 GAL (1–15)+NTX combination modified the mRNA expression of dopamine receptors in VTA and NAc in male and female isolated rats

We have analyzed the mRNA expression of dopamine receptors D1 and D2 in the VTA and NAc in male and female isolated rats.

The administration of GAL (1–15)+NTX and GAL (1–15) alone in male rats is associated with a significant increase in D1 mRNA levels (one-way ANOVA, F_3,20_ = 5.38, p = 0.007, p ≤ 0.05; Table 1) in VTA. In isolated female rats, the combination GAL (1–15)-NTX was associated with increased D1 receptor expression (one-way ANOVA, F_3,18_ = 3.08, p = 0.05, p ≤ 0.05; Table 1).

**TABLE 1:** Effect of the combination of GAL (1–15) and NTX on D1, D2, GALR1, GALR2, GALR3 and 5HT1A mRNA expression in the VTA in a social isolation model in male and female Sprague-Dawley rats.

VTA	Male ♂				Female ♀			
​	Saline + aCSF	Saline + GAL (1–15) 0.3 nmol	NTX 0.3 mg/kg + aCSF	NTX 0.3 mg/kg + GAL (1–15) 0.3 nmol	Saline + aCSF	Saline + GAL (1–15) 0.1 nmol	NTX 0.3 mg/kg + aCSF	NTX 0.3 mg/kg + GAL (1–15) 0.1 nmol
D1	0.7 ± 0.2	1.8 ± 0.4^a^	1.0 ± 0.1	2.0 ± 0.4*	1.3 ± 0.3	5.8 ± 0.5	5.1 ± 1.5	7.2 ± 2.4*
D2	0.8 ± 0.1	0.7 ± 0.1	1.2 ± 0.0*	2.2 ± 0.5**	0.8 ± 0.1	1.9 ± 0.3*	1.4 ± 0.3^a^	2.6 ± 0.5**
GALR1	0.8 ± 0.1	1.0 ± 0.2	0.8 ± 0.1	1.1 ± 0.2	0.9 ± 0.1	1.6 ± 0.2	1.2 ± 0.3	1.5 ± 0.3
GALR2	1.4 ± 0.2	2.0 ± 0.5	1.5 ± 0.4	3.2 ± 0.6*	1.0 ± 0.1	0.9 ± 0.1**	1.1 ± 0.1	1.4 ± 0.1*
GALR3	0.7 ± 0.1	0.9 ± 0.1	0.8 ± 0.1	0.6 ± 0.1	1.9 ± 0.5	3.4 ± 0.6	2.8 ± 0.5	1.3 ± 0.4*
5HT1A	1.2 ± 0.3	1.7 ± 0.3	1.1 ± 0.3	2.2 ± 0.4	1.1 ± 0.1	1.8 ± 0.2*	2.0 ± 0.3**	1.5 ± 0.1

Measures were taken 1 hour after the ICV, injection. Male and female rats: saline + aCSF (n = 5–6), GAL (1–15) group (n = 5–6), NTX, group (n = 5–6), and GAL (1–15)+NTX, group (n = 5–6). Male: Data represent a mean ± standard error of the mean of mRNA, expression in male rats of: D1 (*ap ≤ 0.05 vs. saline + aCSF, and NTX, 0.3 mg/kg + aCSF, groups). D2 (*p ≤ 0.05 vs. NTX, 0.3 mg/kg + GAL (1–15) 0.3 nmol group, **p < 0.01 vs. saline + aCSF, and saline + GAL (1–15) 0.3 nmol groups). GALR2 (*p ≤ 0.05 vs. saline + aCSF, and NTX, 0.3 mg/kg + aCSF, groups). There are no differences between the experimental groups in GALR1, GALR3 and 5HT1A. Female: Data represent a mean ± standard error of the mean of mRNA, expression in female rats of: D1 (*p ≤ 0.05 vs. saline + aCSF, group). D2 (*p ≤ 0.05 vs. saline + aCSF, group, ap ≤ 0.05 vs. NTX, 0.3 mg/kg + GAL (1–15) 0.1 nmol group, **p < 0.01 vs. saline + aCSF, group). GALR2 (*p ≤ 0.05 vs. saline + aCSF, and NTX, 0.3 mg/kg + aCSF, groups, **p < 0.01 vs. NTX, 0.3 mg/kg + GAL (1–15) 0.1 nmol group). GALR3 (*p ≤ 0.05 vs. saline + GAL (1–15) 0.1 nmol group). 5HT1A (*p ≤ 0.05, **p < 0.01 vs. saline + aCSF, group). There are no differences between the experimental groups in GALR1. All comparisons follow a one-way ANOVA, followed by the Newman-Keuls multiple comparisons test. Abbreviations: GAL (1–15) = Galanin (1–15); NTX , naltrexone; D1 = dopamine D1 receptor; D2 = dopamine D2 receptor; GALR1 = galanin receptor 1; GALR2 = galanin receptor 2; GALR3 = galanin receptor 3; 5HT1A = serotonin 1A receptor; VTA , ventral tegmental area; ICV , intracerebroventricular; aCSF , artificial cerebrospinal fluid.

In VTA, the analysis of D2 receptor expression (Table 1) showed that the coadministration of GAL (1–15)+NTX at subthreshold doses increased D2 gene expression in male rats (one-way ANOVA, F_3,18_ = 6.70, p = 0.003, p < 0.05; Table 1). In female rats, a similar effect was observed (one-way ANOVA, F_3,17_ = 5.17, p = 0.010; Table 1), with higher D2 gene expression in the GAL (1–15)+NTX group compared with the control (p < 0.01) and the NTX group (p ≤ 0.05).

In NAc, the combination of GAL (1–15)+NTX lacked an effect on the D1 mRNA expression in male rats (Table 2). However, in isolated female rats, the GAL (1–15) 0.3 nmol and NTX 0.3 mg/kg are significantly associated with an increase in D1 gene expression compared with the control and GAL (1–15) groups (one-way ANOVA, F_3,14_ = 12.05, p = 0.0004, p < 0.01; Table 2).

**TABLE 2:** Effect of the combination of GAL (1–15) and NTX on D1, D2, GALR1, GALR2, GALR3 and 5HT1A mRNA expression in the NAc in a social isolation model in male and female Sprague-Dawley rats.

NAc	Male ♂				Female ♀			
​	Saline + aCSF	Saline + GAL (1–15) 0.3 nmol	NTX 0.3 mg/kg + aCSF	NTX 0.3 mg/kg + GAL (1–15) 0.3 nmol	Saline + aCSF	Saline + GAL (1–15) 0.1 nmol	NTX 0.3 mg/kg + aCSF	NTX 0.3 mg/kg + GAL (1–15) 0.1 nmol
D1	0.3 ± 0.2	0.1 ± 0.0	0.1 ± 0.0	0.3 ± 0.1	2.9 ± 0.9	8.2 ± 3.0	49.9 ± 14.0**	52.8 ± 9.6^aa^
D2	0.5 ± 0.2	0.1 ± 0.0**	0.1 ± 0.0^a^	0.1 ± 0.0*	3.2 ± 0.9	4.4 ± 1.9	13.5 ± 4.0*	22.0 ± 2.9***
GALR1	1.0 ± 0.1	1.1 ± 0.3	0.9 ± 0.1	1.1 ± 0.0	0.8 ± 0.1	0.6 ± 0.1	0.8 ± 0.2	0.7 ± 0.2
GALR2	1.0 ± 0.1	0.9 ± 0.1	0.8 ± 0.1	1.2 ± 0.3	0.7 ± 0.1	0.8 ± 0.1	0.9 ± 0.0	0.9 ± 0.1
GALR3	1.0 ± 0.1	1.5 ± 0.2	0.8 ± 0.2*	1.1 ± 0.1	0.6 ± 0.2	0.8 ± 0.3	0.8 ± 0.1	0.8 ± 0.3
5HT1A	0.9 ± 0.1	1.4 ± 0.3	0.9 ± 0.0	1.1 ± 0.1	0.7 ± 0.1	1.0 ± 0.1	1.1 ± 0.2	0.7 ± 0.1

Measures were taken 1 hour after the ICV, injection. Male and female rats: saline + aCSF (n = 4–6), GAL (1–15) group (n = 4–6), NTX, group (n = 4–6), and GAL (1–15)+NTX, group (n = 4–6). Male: Data represent a mean ± standard error of the mean of mRNA, expression in male rats of: D2 (*^,a^ p ≤ 0.05, **p < 0.01 vs. saline + aCSF, group), GALR3 (*p ≤ 0.05 vs. saline + GAL (1–15) 0.3 nmol group). There are no differences between the experimental groups in D1, GALR1, GALR2 and 5HT1A. Female: Data represent a mean ± standard error of the mean of mRNA, expression in female rats of: D1 (**^, aa^p <0.01 vs. saline + aCSF, and saline + GAL (1–15) 0.1 nmol groups). D2 (*p ≤ 0.05 vs. the rest of the groups; ***p < 0.001 vs. saline + aCSF, and saline + GAL (1–15) 0.1 nmol groups). There are no differences between the experimental groups in GALR1, GALR2, GALR3 and 5HT1A. All comparisons follow a one-way ANOVA, followed by the Newman-Keuls multiple comparisons test. Abbreviations: GAL (1–15) = Galanin (1–15); NTX , naltrexone; D1 = dopamine D1 receptor; D2 = dopamine D2 receptor; GALR1 = galanin receptor 1; GALR2 = galanin receptor 2; GALR3 = galanin receptor 3; 5HT1A = serotonin 1A receptor; NAc , nucleus accumbens; ICV , intracerebroventricular; aCSF , artificial cerebrospinal fluid.

In NAc, administration of GAL (1–15)+NTX was associated with decreased D2 gene expression in male rats (one-way ANOVA, F_3,17_ = 4.69, p = 0.014; p < 0.05; Table 2), while in female rats, this combination induced an increase of D2 mRNA expression (one-way ANOVA, F_3,15_ = 13.45, p = 0.0002, p ≤ 0.05; Table 2) compared with the rest of the groups.

#### 3.6.4 GAL (1–15)+NTX combination changed the mRNA expression of GAL receptors in VTA and NAc in male and female isolated rats

In the VTA, the administration of GAL (1–15)+NTX in male rats is associated with a significant increase in GALR2 expression (one-way ANOVA, F_3,19_ = 3.23, p = 0.046; p ≤ 0.05; Table 1) compared with the control and NTX groups. In female rats, the same treatment also led to an increase in GALR2 expression (one-way ANOVA, F_3,18_ = 4.74, p = 0.01, p ≤ 0.05; Table 1), and decreased GALR3 expression (one-way ANOVA, F_3,17_ = 3.41, p = 0.042, p ≤ 0.05; Table 1), compared with the GAL (1–15) alone group.

Expression of GAL receptors was also analysed in NAc. We observed that GAL (1–15)+NTX coadministration did not affect the GAL receptor expression (Table 2), neither in male nor female rats, in the NAc nuclei.

Finally, the administration of GAL (1–15)+NTX had no effect on 5-HT1A expression in either male or female rats in the two areas analyzed (Tables 1, 2).

## 4 Discussion

In this study, we demonstrate that social isolation during critical developmental periods increased alcohol consumption and alcohol seeking within the respective male and female experimental cohorts. Importantly, we provide the first evidence that the combination of GAL (1–15) with a mu-opioid receptor antagonist such as NTX under conditions of social isolation is associated with a reduction of the voluntary alcohol intake in male rats, as well as reducing alcohol seeking in an operant self-administration model in rats of both sexes. Furthermore, alterations in the expression of mesolimbic-related markers may contribute to the behavioral actions of GAL (1–15)+NTX, as suggested by changes in C-Fos and mu-opioid receptor (MOR) mRNA expression in the VTA and NAc, as well as by alterations in the expression of dopaminergic and galaninergic genes, supporting a possible interaction between GAL (1–15) and the opioid system.

In the present investigation, we report consistent evidence that the combination of GAL (1–15) and NTX reduces alcohol-seeking behavior in male and female rats. In contrast, reductions in alcohol consumption were observed only in males under social isolation conditions. Together, these findings provide preclinical evidence supporting additional investigation of the interaction between GAL (1–15) and NTX in alcohol-related behaviors. However, studies are still needed to assess the translational relevance and therapeutic potential of this combination.

In the current work, we used a social isolation model in which male and female rats were housed individually for 6 weeks starting at 21 days of age, corresponding to the adolescence period (McCool and Chappell, 2009). In our study, we used various behavioral tests to assess the effects of social isolation during vulnerable periods, including the EPM and the light-dark test to analyze anxiety-related behaviors, as well as alcohol consumption patterns. It is important to note that the distinct acquisition protocols used for males and females preclude a direct statistical comparison between the sexes. These specific procedures were implemented solely to facilitate optimal adaptation to alcohol consumption within each cohort. While the final behavioral endpoints assessed in both sexes were identical (namely, the two-bottle choice and operant self-administration tests) and no relevant changes were observed during training in either sex, we acknowledge that these distinct behavioral histories represent an interpretational limitation. Consequently, direct cross-sex comparisons should be approached with mindfulness regarding these baseline differences.

Overall, these results suggest that the effects of the social isolation model require careful evaluation, as the observed changes appear to primarily affect locomotor and exploratory parameters rather than consistent indicators of anxiety. In this regard, while non-isolated females showed reductions in locomotor speed and distance travelled in the OFT, no significant differences were observed in the light-dark test in males, a finding also reported in previous studies using social isolation paradigms (McCool and Chappell, 2009).

Within their respective experimental paradigms, both sexes showed a significant reduction in alcohol consumption and alcohol-seeking behavior, consistent with previous studies (Butler et al., 2014). These findings indicate that alcohol-related behaviors were successfully established under the experimental conditions employed. However, these methodological variations in behavioral history must remain a central consideration when evaluating and contrasting the outcomes between cohorts.

Early-life social isolation increases the risk of developing alcohol-related disorders in adulthood (Wardell et al., 2020; Le et al., 2021), while social support acts as a protective factor (Akerlind and Hörnquist, 1992; Holt-Lunstad et al., 2017; Walsh et al., 2024). The COVID-19 pandemic highlighted this link, with isolation associated with greater distress and alcohol use (Charles et al., 2021; Kilian et al., 2022; Fendrich et al., 2024). Animal studies confirm that isolation during critical developmental periods leads to heightened alcohol intake and motivation (McCool and Chappell, 2009; Chappell et al., 2013; Butler et al., 2014; Lesscher et al., 2015). Together, these findings highlight the translational value of the social isolation model for studying the neurobiological mechanisms underlying increased vulnerability to AUD and for evaluating potential therapeutic interventions.

This study aimed to evaluate whether the combination of GAL (1–15) with NTX can influence alcohol consumption and seeking in adulthood following a period of social isolation during early developmental stages, in both male and female rats.

First, the effect of the combination of GAL (1–15)-and NTX in the alcohol consumption was studied, using the two-bottle choice paradigm, the most well-known variant of voluntary consumption models (Leeman et al., 2010), in rats under social isolation. We observed that the combination of GAL (1–15) and NTX reduced alcohol consumption in male rats, with a decrease observed at 2 h. These results are in agreement with our previous work, where this combination induced a reduction of alcohol consumption in males without isolation (Flores-Gómez et al., 2025).

For the first time, we evaluated the effect of GAL (1–15) alone in female rats in a voluntary alcohol intake using the two-bottle choice test in a social isolation model. We found that GAL (1–15) induced a significant 50% reduction in ethanol consumption at 2 h. In previous studies, we demonstrated that GAL (1–15) also induced a marked decrease in ethanol preference and consumption in non-isolated male rats in this test (Millón et al., 2019). These findings reinforce the evidence that GAL (1–15) alone is effective in reducing alcohol consumption in both sexes, under different conditions. Interestingly, GAL (1–15) exhibited a non-monotonic dose-response profile in the two-bottle choice test, where only the intermediate dose (3 nmol) significantly reduced ethanol intake, whereas the highest dose (6 nmol) was ineffective. Such inverted U-shaped or bell-shaped dose-response curves are a common characteristic of neuropeptide signaling (Díaz-Cabiale et al., 2014; Millón et al., 2014; Millón et al., 2016). Indeed, this biphasic phenomenon has been previously reported for GAL (1–15) in other physiological contexts, as exemplified by its reported effects on anxiety-like behaviors or its functional interaction with 5-HT1A receptors (Millón et al., 2014; Millón et al., 2016).

This non-monotonic pattern is frequently attributed to complex receptor dynamics. At intermediate concentrations, GAL (1–15) optimally facilitates the activation or formation of specific galanin receptor complexes, such as GALR1–GALR2 heteromers. However, higher concentrations (e.g., 6 nmol) may trigger counter-regulatory mechanisms. These include the rapid desensitization and endocytosis of the active receptor complexes, or a shift toward alternative, lower-affinity signaling pathways that functionally counteract the initial response. Therefore, the behavioral effects of GAL (1–15) are tightly restricted to a narrow, optimal concentration range (Millón et al., 2014; Millón et al., 2016).

However, the combination of GAL (1–15) and NTX at the doses tested did not produce significant effects on alcohol consumption in socially isolated female rats. A primary limitation of this study involves the subthreshold dosing strategy used to detect synergy. Because female rats exhibit high individual threshold doses for both GAL (1–15) and NTX, the subthreshold doses selected here may have been too low to trigger a combined therapeutic response in this specific cohort. While these distinct dosing requirements might tentatively suggest underlying sex-dependent variations in response to GAL (1–15)+NTX administration, the methodological differences in the acquisition procedures between males and females prevent definitive conclusions. Therefore, these potential variations should be considered purely speculative. Nonetheless, they raise the hypothesis of a possible role for sexual dimorphic mechanisms within the galaninergic system. This is supported by our recent work, which revealed sex-specific neurobiological differences in the expression and regulation of galaninergic components (Cantero-García et al., 2026) warranting further investigation under fully standardized procedural conditions.

Notably, during the two-bottle choice tests, reduced water consumption was observed in female rats receiving NTX, including both the NTX-alone and GAL (1–15)-NTX coadministration groups. In this regard, NTX has been shown to reduce water intake in specific contexts, such as deprivation or choice paradigms (Frenk and Rosen, 1979). These effects have been attributed to nonspecific mechanisms, such as aversive properties or changes in the distribution of intake. Consistent with these findings, a reduction in total fluid intake was also observed in females, whereas no significant changes in ethanol consumption were detected. Furthermore, given that GAL (1–15) was administered at subthreshold doses in the combined treatment, the observed effects on fluid intake are likely primarily mediated by NTX rather than by a specific effect of the GAL (1–15)-NTX combination on alcohol consumption.

To further explore the combination of GAL (1–15)-NTX under social isolation, we used the alcohol self-administration, a well-established model for assessing drug-seeking behavior (Sanchis-Segura and Spanagel, 2006; Ettenberg, 2009). We found that the GAL (1–15)-NTX combination induced a substantial reduction in alcohol behavior in both male and female cohorts, suggesting a loss of alcohol-induced motivational drive. In this sense, the combination of GAL (1–15) and NTX in animals that have suffered a period of social isolation is effective in reducing the seeking and motivation for alcohol, which highlights the importance of this combination in AUDs in vulnerable conditions. It is not only effective under these conditions, but also in rats without social isolation, as shown in previous studies (Flores-Gómez et al., 2025).

GAL (1–15) effectively reduces alcohol-seeking behavior in male rats, with 1 nmol being the most effective dose in an operant self-administration model (Cantero-García et al., 2022b). The present study further demonstrates that GAL (1–15) reduces alcohol-seeking behavior in a social isolation model in both sexes, thus expanding the evidence for the role of this peptide in alcohol-related behaviors. However, in isolated females, the effective dose identified was lower than that previously found in males. These apparent dose differences must be interpreted with caution. First, the distinct acquisition and training procedures used between males and females preclude a direct comparison of these effective doses. Second, in the absence of pharmacokinetic (PK) and pharmacodynamic (PD) measurements, it remains unknown whether these variations reflect differences in drug exposure, metabolism, brain penetration, or actual target engagement. Therefore, these dosing differences cannot be definitively attributed to differential biological sensitivity or sex-dependent mechanisms at this stage. Nonetheless, they raise the hypothesis of potential sex-specific variations within the galaninergic system. While recent work indicates a baseline sexual dimorphism in the expression and regulation of galaninergic components (Cantero-García et al., 2026) future studies combining standardized behavioral paradigms with PK/PD assessments are required to test this hypothesis. Since the effects of GAL (1–15) in non-isolated females have not yet been evaluated, future studies should also address this question to determine whether social isolation modulates the peptide’s response.

Notably, the lower dose combination reduced operant alcohol-seeking in females, whereas the higher dose combination failed to alter voluntary two-bottle choice consumption. This disparity likely reflects the distinct neurobiological mechanisms driving these paradigms: two-bottle choice measures free-choice intake, while operant self-administration evaluates goal-directed motivation. Although both paradigms involve reward-related circuitry, operant self-administration primarily assesses the motivational component of alcohol-directed behavior. In contrast, the two-bottle choice test evaluates voluntary alcohol consumption under free-access conditions. These differences may contribute to the distinct dose-dependent effects observed in the present study.

In the social isolation model, the GAL (1–15)-NTX combination was associated with increased C-Fos mRNA expression in the VTA and NAc of both male and female rats. Although C-Fos is widely used as a marker of neuronal activation, the observed increase should be interpreted as an indirect indicator of neuronal activity within these regions. These transcriptional changes were accompanied by elevated D1 receptor expression and modulation of D2 receptor levels in these regions. Given the critical role of the VTA and NAc in alcohol reinforcement and addiction, these findings suggest that GAL (1–15)+NTX may influence key dopaminergic pathways involved in alcohol-related behaviors.

Interestingly, GALR2 expression was increased in the VTA following GAL (1–15)-NTX administration in both male and female rats. This finding is consistent with previous evidence identifying GALR2 as a key component of the GALR1-GALR2 heteroreceptor complex (Borroto-Escuela et al., 2014; Millón et al., 2014) which is proposed to mediate the biological actions of GAL (1–15). Therefore, the consistent upregulation observed in both sexes supports the involvement of GALR2 in the effects induced by the combined treatment.

Importantly, only the GAL (1–15)-NTX combination increased MOR expression in isolated female rats in the VTA and NAc, and a similar trend was observed in males in these regions, suggesting a possible involvement of the opioid system. Dopamine release in the VTA is partially enhanced by stimulation of MOR located on inhibitory GABAergic interneurons within this region (Johnson and North, 1992; Di Chiara, 1998; Berrettini, 2016). Based on these findings, the observed changes in MOR mRNA expression may reflect transcriptional adaptations within the opioid system. However, it should also be considered that the increase in MOR mRNA expression observed following GAL (1–15)-NTX administration may be influenced by compensatory responses associated with the administration of MOR antagonists such as NTX. In addition, NTX administration alone did not produce the same increase in MOR expression observed with the combined administration of GAL (1–15) and NTX, suggesting that the interaction between the two compounds may contribute to these effects. Therefore, further studies are required to explore better the mechanisms underlying these effects.

Previous studies suggested that the GAL (1–15)-NTX combination could modulate the dopaminergic system in the VTA and NAc under non-isolated conditions (Flores-Gómez et al., 2025). The present study observed similar effects in the social isolation model, supporting a possible role for mesolimbic-related molecular changes in this model. Recent studies indicate the presence of MOR-GALR1 heteromers in VTA, highlighting GPCR heteromers as potential targets for drug development (Moreno et al., 2017; Cai et al., 2019). Therefore, the action of GAL (1–15) in isolation could imply the existence of a GALR1-GALR2-MOR complex, an interaction that requires further investigation.

Importantly, the molecular analyses performed in the present study were limited to analyzing mRNA expression. Therefore, these findings should not be interpreted as direct evidence of corresponding changes in protein expression, receptor availability, or receptor function. Future studies incorporating protein quantification and functional approaches are necessary to determine the biological significance of these transcriptional alterations.

Taken together, these findings suggest that the dopaminergic and mesolimbic opioid systems may contribute to the effects observed after GAL (1–15)-NTX administration in the social isolation model. However, further studies are needed to explore these results in greater depth and to understand better the mechanisms underlying the effect of the GAL (1–15)-NTX combination on alcohol consumption and seeking behavior. Additionally, the relatively small sample sizes in the molecular analyses may limit statistical power and should be considered when interpreting these findings.

In conclusion, early social isolation has been shown to produce a moderate increase in alcohol intake and alcohol seeking. In adulthood, the GAL (1–15)-NTX combination reduced alcohol-seeking behavior in both sexes and decreased alcohol consumption in males, even in this social isolation model. This effect appears to be associated with the mesolimbic system, highlighting an interaction of GAL (1–15) with the opioid system. These findings suggest that the GAL (1–15)+NTX combination could be further explored as a promising pharmacological strategy for AUD under conditions of increased vulnerability.

## 5 Limitations

This study has some limitations that should be considered when interpreting the results. These include the lack of protein-level analysis, the absence of functional experiments to establish causality, the use of different behavioral procedures between sexes, and the assessment of molecular changes at a single point in time. Future studies are necessary to address these issues.

## Data Availability

The raw data supporting the conclusions of this article will be made available by the authors, without undue reservation.
