# Supplementary material for: Early socially isolated rats of both sexes reduce alcohol consumption and seeking by the Galanin (1–15) and naltrexone combination
Source: Front Pharmacol. 2026 Sep 18;17:1836976. doi: 10.3389/fphar.2026.1836976 (PMC13630733; doi:10.3389/fphar.2026.1836976)
Supplement: Supplementary file 1 [file DataSheet1.pdf]

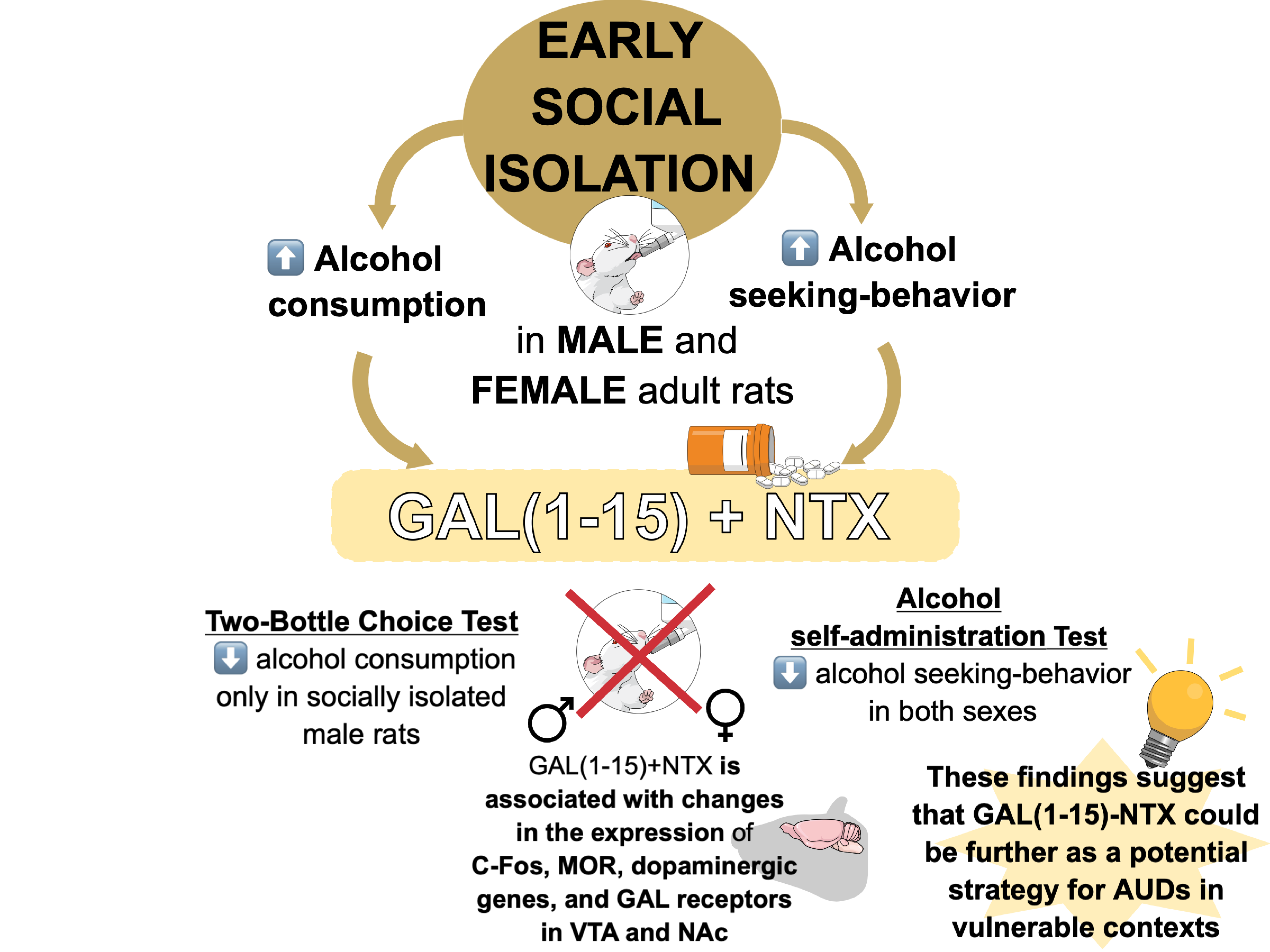
EARLY SOCIAL ISOLATION
Alcohol consumption
Alcohol seeking-behavior
in MALE and FEMALE adult rats
GAL(1-15) + NTX
Two-Bottle Choice Test
alcohol consumption only in socially isolated male rats
Alcohol self-administration Test
alcohol seeking-behavior in both sexes
GAL(1-15)+NTX is associated with changes in the expression of C-Fos, MOR, dopaminergic genes, and GAL receptors in VTA and NAc
These findings suggest that GAL(1-15)-NTX could be further as a potential strategy for AUDs in vulnerable contexts
