# Supplementary material for: Early socially isolated rats of both sexes reduce alcohol consumption and seeking by the Galanin (1–15) and naltrexone combination
Source: Front Pharmacol. 2026 Sep 18;17:1836976. doi: 10.3389/fphar.2026.1836976 (PMC13630733; doi:10.3389/fphar.2026.1836976)
Supplement: Supplementary file 2 [file Table1.docx]

Supplementary Material

**Stereotaxic surgery**

This protocol has been described previously (Diaz-Cabiale et al., 2011; Millón et al., 2015; Cantero-García et al., 2022). Briefly, the rats were anaesthetized with Isoflurane (ISOFLO® ZOETIS ABOTT Liquid gaseous anaesthetic, 100% Isoflurane), 5% in the induction chamber, and maintenance of 1.5-2.5% Isoflurane mask and placed in the stereotactic instrument. A chronic 22-gauge stainless-steel guide cannula was stereotaxically implanted into the right lateral cerebral ventricle (+1.4 mm lateral, -1 mm posterior to bregma, and 3.6 mm below the surface of the skull) (Paxinos, 1986). Animals were individually housed after surgery and had a recovery period of 7 days before the behavioral test.

**Administration of substances and drugs**

The intracerebroventricular injections in the lateral ventricle were performed using a 26-gauge stainless-steel injection cannula connected via a PE-10 tubing to a Hamilton syringe. The total volume was 5 μl per injection, and the infusion time was 1 minute with special care that during the process, the dilutions were administered fluently and without overflowing. Solutions were prepared freshly, and the peptides were dissolved in artificial cerebrospinal fluid (composition is 120nM NaCl, 20nM NaH_2_CO_3_, 2nMKCl, 0.5nM KH_2_PO_4_,1.2nM CaCl_2_, 1.8 nM MgCl_2_, 0.5 nM Na_2_SO_4_, and 5.8 nM D-glucose, pH 7.4).

For subcutaneous administration of NTX, the test dose was 0.3-1-3-6 mg/Kg and was administered in a 2 ml/Kg body weight volume. The doses employed and the injection protocol were based on those of previous studies (Maggio et al., 2018; Nieto et al., 2018; Flores-Gómez et al., 2025).

**Light-Dark test**

The light-dark box apparatus consisted of two chambers (26x25x27 cm) joined lengthwise. One chamber was black, and the other was white. The white chamber was illuminated by a 40W light bulb. Animals were individually placed in the lightbox's centre and allowed to explore freely for 5 minutes. After each trial, all surfaces were cleaned with a paper towel and 70% ethanol solution. The latency to enter (with all four paws) in the dark chamber and the time in the light chamber were recorded (Millón et al., 2015).

**Two-bottle choice training**

The animals were pretrained to drink alcohol before the two-bottle choice test. In isolated male rats, and to achieve high alcohol consumption values, the animals received forced access to 10% ethanol for three days, followed by four days of free choice between ethanol and water (McCool and Chappell, 2009). In experiment 2, with isolated female rats, a previously described training gradual protocol was followed (Millón et al., 2019). Animals first had access to water in both bottles for 7 days, then choose between water and increasing ethanol concentrations (3%, 6%, and 10%) for 7 days each, followed by a choice between 10% ethanol and water until intake stabilized.

After each training period, the treatments were administered and the two-bottle choice test was performed.

**Operant self-administration apparatus and training methods**

Training and testing were conducted in standard operant chambers (Panlab, Barcelona, Spain). Each chamber was equipped with a drinking reservoir positioned 4 cm above the grid floor in the centre of the front panel of the chamber, and two retractable levers were located 3 cm to the right and left of the drinking receptacle.

Each reservoir was calibrated to dispense 0.1 ml when the fixed ratio of lever presses was obtained. Chambers were connected to a computer running Packwin Sofware (2.0, Panlab, Barcelona, Spain), which was used to automatically record the number of active and inactive lever responses and the number of reinforcement for each rat in each session (Cantero-García et al., 2022a,b).

To assess the impact of social isolation on alcohol motivation, the breakpoint was measured in male isolated rats using a progressive ratio schedule, where the response requirement increased after each reward (Cantero-García et al., 2022a). During a one-hour session, the maximum ratio value (breaking point), completed receiving a single reward of alcohol, was measured and considered an index of motivation.

**Alcohol self-administration test**

In isolated female rats, the self-administration behavior was induced by a two-step paradigm: First, rats were exposed to intermittent access to 10% alcohol and 0.2% saccharin for 3 weeks to facilitate the acquisition of a high level of alcohol intake (Alén et al., 2009; Lebourgeois et al., 2018) with minor modifications. Rats were given access to two bottles, one bottle containing tap water and the other containing 10% alcohol and 0.2% saccharin, for 24 hours sessions on Mondays, Wednesdays, and Fridays for 3 weeks (a total of nine drinking sessions). At the end of each session, bottles were weighed to assess alcohol consumption (g ethanol/Kg body weight) and preference (the ratio of alcohol consumed to total fluid intake). The bottle placement in the cage (left or right) was alternated between sessions to avoid side preferences. Before self-administration, rats were placed on a water restriction schedule for 2-4 days to facilitate training in lever pressing. The rats were initially trained to self-administer 10% alcohol and 0.2% saccharin, decreasing the saccharin percentage over the days and maintaining that of alcohol until the animals reached a stable level of 10% alcohol responding.

In isolated male rats, a modified protocol was employed. Following the two-bottle ethanol test, animals were directly trained to self-administer 10% alcohol, without the saccharin fading procedure, as they already displayed stable levels of alcohol consumption.

**Open Field Test**

Animals were individually placed and allowed to explore freely in the open field (100x100x50 cm). Their behavior was recorded over 5 minutes by a ceiling-mounted video camera, and locomotor activity was analyzed using the video-tracking software Ethovision XT (Noldus, S.L). After each trial, all surfaces were cleaned with a paper towel and 70 percent ethanol solution. After each trial, all surfaces were cleaned with a paper towel and 70% ethanol solution. Nº entries in the centre, time in entries, total distance travelled, and mean speed were recorded (Flores-Gómez et al., 2025).

**Elevated Plus-Maze**

The maze is 40 cm above the floor and has cross-shaped platforms, two open arms with 2 cm high plexiglass sides, and two closed arms with 40 cm high opaque walls. Rats were placed on the central platform facing an open arm and allowed to explore the maze for 5 minutes. Entry into the open or closed arm was defined as entering with all four paws. After each trial, all surfaces were cleaned with a paper towel and 70% ethanol solution. Percent entries and time in open arms, total distance (cm), and mean speed (cm/s) were recorded (Cantero-García et al., 2022b).

***VTA* and *NAc* dissections**

Isolated male and female rats from the alcohol self-administration procedures were killed by decapitation 1 hour after administration of the treatments, and the brains were rapidly removed and frozen until their use. The nuclei dissections were conducted as described (Garcia-Perez et al., 2016) with modifications. Brains were sliced on the brain matrix (1 mm) and kept at -20ºC until each region of interest came into the cutting plane. For VTA analysis, one 1 mm coronal brain section was obtained from bregma -6.04 mm (Paxinos, 1986). For the NAc study, two consecutive 1 mm coronal slices were made corresponding to approximate bregma +1.2 mm (Paxinos, 1986). Tissues of interest were dissected using a 2 mm internal diameter punching device. Bilateral NAc and VTA punches were collected into Eppendorf tubes.

**RNA isolation and quantitative real-time polymerase chain reaction analysis**

Total RNA was isolated from the VTA and NAc punches using a RNeasy Lipid Tissue Kit (Qiagen, Hilden, Germany). cDNA was obtained using a Reverse Transcriptase Core Kit (Eurogentec, Lieja, Belgium). These steps were performed according to the manufacturer’s instructions.

All PCR were conducted in triplicate using FastStart essential DNA green master (Roche Diagnostics GmbH, Manncheim, Germany) in LigtCycler 96 system (Roche Diagnostics GmbH, Manncheim, Germany). The data were analyzed using the comparative Ct method and normalized to measures of glyceraldehyde- 3-phosphate dehydrogenase (GAPDH) mRNA.

The primer sequences used in this study are:

**Table S1.** Oligonucleotide primers used in q-PCR

| **Gene** | **Forward primer (5´-3´)** | **Reverse primer (5´-3´)** |
| --- | --- | --- |
| ***C-Fos*** | AAACGGAGAATCCGAAGG | CGTCTTCAAGTTGATCTGTC |
| ***MOR*** | CTAACCACCAGCTAGAAAATC | TTTGAATGCAGGATCAGATG |
| ***D1*** | AAACAAATAGGCCAAGAGTC | CTTTCCAATCTGAAAACTGC |
| ***D2*** | AAGCGCCGAGTTACTGTCAT | GACCACAAAGGCAGGGTTG |
| ***GALR1*** | AAAACTGGACAAAACTTAGCC | GGATACCTTTGTCTTTGCTC |
| ***GALR2*** | AACAGGAATCCACAGACC | CCCTTTGGTCCTTTAACAAG |
| ***GALR3***  ***5HT1A*** | ACAGATCTCTTCATCCTCAAC  AACTATCTCATCGGCTCC | AGATGAGCAGATGTACCG  ACATCCAGGGCGATAAAC |

**SUPPLEMENTAL RESULTS**

**Figure S1.** Effect of Social Isolation in Two-Bottle Choice training in male Sprague–Dawley rats

**A)**

**B)**

**Forced exposure to ethanol**

**Free choice between ethanol and water**

**Figure S1.** Effect of social isolation in Two-Bottle Choice Training, including three days of forced access to 10% ethanol followed by four days of free choice between 10% ethanol and water prior to the test, in male Sprague–Dawley rats. **(A)** Vertical bars represent a mean ± standard error of the mean of EtOH intake (g/Kg) during three days of forced exposure. *p ≤0.05 non-isolated group vs isolated group on the second and third days of exposure, according to a Student t-test. **(B)** Vertical bars represent a mean ± standard error of the mean of EtOH intake (g/Kg) over four days of choice between 10% ethanol and water. There are no significant differences between groups according to a Student t-test.

**Table S2.** Effect of Social Isolation in the Light-Dark Test in male Sprague-Dawley rats

| **MALE ♂** | | **Light-Dark Test** | |  |
| --- | --- | --- | --- | --- |
| **Parameters**  **Measured** | ***Latency to enter***  ***in dark chamber (s)*** | | ***Time in light***  ***chamber (s)*** | |
| **Non-isolated group** | 18.0 ± 4.3 | | 74.3 ± 7.5 | |
| **Isolated group** | 22.4 ± 2.4 | | 83.6 ± 3.2 | |

**Table S2.** Data represent a mean ± standard error of the mean of latency to enter in dark chamber (s) and time in light chamber (s). Experimental groups: non-isolated group (n=8), isolated group (n=40). There are no differences, according to a Student t-test, between the experimental groups.

**Figure S2.** Effect of Social Isolation in Two-Bottle Choice training in female Sprague–Dawley rats

**Figure S2.** Effect of social isolation in two-bottle choice training during the seven days prior to the test in female Sprague–Dawley rats. Vertical bars represent a mean ± standard error of the mean of EtOH intake (g/Kg). There are no significant differences between groups according to a Student t-test.

**Figure S3.** Effect of social isolation during the training sessions of alcohol self-administration prior to the test in female Sprague–Dawley rats

**Figure S3.** Effect of social isolation on EtOH reinforcements during alcohol self-administration training prior to the test in female Sprague–Dawley rats. Vertical bars represent a mean ± standard error of the mean of EtOH reinforcements. No significant differences were observed between groups according to a Student t-test.

**Table S3.** Effect of Social Isolation in the OFT and EPM in two-bottle choice and alcohol self-administration group in female Sprague-Dawley rats

**Table S3.** Data represent a mean ± standard error of the mean of nº entries, time in entries (s), total distance (cm) and mean speed (cm/s) in OFT and % entries in open arms, % time in open arms, total distance (cm) and mean speed (cm/s) in EPM. Experimental groups: non-isolated group (n=8), isolated group (n=32). OFT in two-bottle choice group: nº entries *p ≤0.05, total distance and mean speed ** ªªp <0.01 vs non-isolated group. EPM in two-bottle choice group: total distance and mean speed ** ªªp <0.01 vs non-isolated group. OFT in alcohol self-administration group: nº entries *p ≤0.05, time in entries **p <0.01 vs non-isolated group. EPM in alcohol self-administration group: % entries in open arms **p <0.01 vs non-isolated group. All comparisons follow a Student t-test.

**Table S4.** Effect of GAL(1-15) administration at 0.1 and 0.3 nmol on active and inactive lever presses in the alcohol self-administration test in isolated female Sprague-Dawley rats

| **Treatment** | **% change of active lever presses compared with respective control** | **% change of inactive lever presses compared with respective control** |
| --- | --- | --- |
| **aCSF** | 100.0 ± 41.3 | 100.0 ± 57.7 |
| **GAL(1-15) 0.1 nmol** | 86.7 ± 21.8 | 47.6 ± 19.1 |
| **aCSF** | 100.0 ± 19.1 | 100.0 ± 64.6 |
| **GAL(1-15) 0.3 nmol** | **43.9 ± 7.4**** | 43.8 ± 43.8 |

**Table S4.** Data represent a mean ± standard error of the mean of the percentage change in active and inactive lever presses in the alcohol self-administration test compared to the respective control. Active lever presses: **p <0.01 control group vs GAL(1-15) 0.3 nmol group, according to a Student t-test. There are no differences between the experimental groups in the inactive lever presses according to a Student t-test.

**Table S5.** Dose-response effects of Naltrexone on active and inactive lever presses in alcohol self-administration test in isolated female Sprague-Dawley rats

| **Treatment** | **% change of active lever presses compared with respective control** | **% change of inactive lever presses compared with respective control** |
| --- | --- | --- |
| **Saline** | 100.0 ± 33.5 | 100.0 ± 61.2 |
| **NTX 0.3 mg/Kg** | 101.4 ± 52.6 | 50.0 ± 50.0 |
| **Saline** | 100.0 ± 32.8 | 100.0 ± 100.0 |
| **NTX 1 mg/Kg** | 45.5 ± 12.7 | 104.2 ± 38.4 |
| **Saline** | 100.0 ± 25.1 | 100.0 ± 48.5 |
| **NTX 3 mg/Kg** | **45.9 ± 14.7*** | 122.4 ± 55.6 |

**Table S5.** Data represent a mean ± standard error of the mean of the percentage change in active and inactive lever presses in the alcohol self-administration test compared to the respective control. Active lever presses: *p ≤0.05 control group vs NTX 3 mg/Kg group, according to a Student t-test. There are no differences between the experimental groups in the inactive lever presses according to a Student t-test.

**Figure S4.** Effect of GAL(1-15) and NTX administration on Total Fluid Intake at 2h in the Two-Bottle Choice Test in a social isolation model in male Sprague–Dawley rats

**Figure S4.** GAL(1-15) and NTX administration effect on total fluid intake in socially isolated male Sprague-Dawley rats. NTX 1 mg/Kg or saline was administrated SC 30 min before the test, and GAL(1-15) 1 nmol or aCSF was administered ICV 15 min before the test. 5-9 animals/ group. Vertical bars represent a mean ± standard error of the mean of total fluid (mL) 2 hours after the two-bottle choice test. There are no differences according to a one-way analysis of variance (ANOVA) between the experimental groups.

**Figure S5.** Effect of GAL(1-15) and NTX administration on Total Fluid Intake at 2h in the Two-Bottle Choice Test in a social isolation model in female Sprague–Dawley rats

**Figure S5.** GAL(1-15) and NTX administration effect on total fluid intake in socially isolated female Sprague-Dawley rats. NTX 1 mg/Kg or saline was administrated SC 30 min before the test, and GAL(1-15) 1 nmol or aCSF was administered ICV 15 min before the test. 7-8 animals/ group. Vertical bars represent a mean ± standard error of the mean of total fluid (mL) 2 hours after the two-bottle choice test. *p ≤0.05 saline + GAL(1-15) 1 nmol vs saline + aCSF group; **p <0.01 saline + GAL(1-15) 1 nmol vs NTX 1 mg/Kg + aCSF and NTX 1 mg/Kg + GAL(1-15) 1 nmol groups, according to a one-way analysis of variance (ANOVA), followed by the Newman-Keuls multiple comparisons test.
